# Multimodal AI for Same-Session Estimation of PHQ-9 Screen-Positive Status from Counselor-Conducted Fixed-Structure Interviews: A Preregistered Internal Validation Study of Incremental Value and Calibration

**DOI:** 10.3390/healthcare14213602

**Published:** 2026-10-23

**Authors:** Keita Kiuchi, Kotaro Kashihara, Toshiki Takanabe, Hidehiro Umehara, Koushi Irizawa, Masahito Nakataki, Shusuke Numata, Xin Kang, Minoru Yoshida, Kazuyuki Matsumoto

**Affiliations:** 1 National Institute of Occupational Safety and Health, Japan Organization of Occupational Health and Safety, Kawasaki, Kanagawa 214-8585, Japan; 2 Works Applications, Chiyoda, Tokyo 102-0083, Japan; 3 Graduate School of Science and Technology, Tokushima University, Tokushima 770-8506, Japan; 4 Health Service, Counseling and Accessibility Center, Tokushima University, Tokushima 770-8502, Japan; 5 Department of Psychiatry, Graduate School of Biomedical Science, Tokushima University, Tokushima 770-8503, Japan; 6 Graduate School of Technology, Industrial and Social Sciences, Tokushima University, Tokushima 770-8506, Japan

**Keywords:** multimodal artificial intelligence, affective computing, digital mental health, depression screening, PHQ-9, fixed-structure mental health interviews, Gaussian mixture model, probability calibration, internal validation, emotion- and topic-eliciting interview phases

## Abstract

**Highlights:**

**What are the main findings?**

- In this cohort comprising adult and pediatric participants, the preregistered native-scale FA+GMM model had less favorable mean performance estimates than FA; post hoc training-fold standardization attenuated this difference, but the standardized GMM summaries showed no clear incremental value beyond participant-level mean factor scores.
- In the primary threshold-10 analysis, the design-only benchmark had the most favorable mean point estimates across repeats; the interview-feature models had low calibration slopes for cross-fitted recalibrated probabilities; and exploratory fixed-summary and length-stability estimates varied across interview phases and tested retained-segment conditions.

**What are the implications of the main findings?**

- For AI-based depression assessment in healthcare, complex representations should demonstrate incremental value over simpler, traceable representations and explicit design-aware benchmarks, with calibration assessed alongside discrimination.
- Interview phase and segment-retention criteria should be prespecified as validation dimensions; the present analyses did not establish an optimal phase, retention rule, or required input duration.

**Abstract:**

**Background/Objectives:** Multimodal mental health assessment integrates acoustic, facial, and linguistic signals. We tested whether Gaussian mixture model (GMM) posterior summaries added value beyond participant-level factor analysis (FA) means for same-session estimation of Patient Health Questionnaire-9 (PHQ-9) screen-positive status. **Methods:** This preregistered secondary analysis included counselor-conducted fixed-structure interview results from 103 participants (83 adults, 20 pediatric participants; 24 screen-positive). The pediatric outcome was a study-specific operational label because criterion validity was not established for the administered form and cutoff. Repeated nested cross-validation compared FA+GMM and FA, both including a cohort indicator, with a design-only benchmark. Average precision (AP) was the primary metric; the no-skill baseline was 24/103 = 0.233. **Results:** In the primary analysis, mean AP was 0.302 for FA, 0.282 for native-scale FA+GMM, and 0.332 for the design-only benchmark. Mean paired FA+GMM-minus-FA differences were −0.020 for AP, −0.031 for the area under the receiver operating characteristic curve, and +0.0016 for Brier score. Conditional bootstrap comparisons with the design-only benchmark were imprecise. Calibration slopes after cross-fitted recalibration were 0.104 for FA and 0.093 for FA+GMM. Post hoc training-fold standardization increased FA+GMM AP to 0.300, close to FA. Refitting with a pediatric threshold of 11 did not alter the incremental-value conclusion. **Conclusions:** GMM summaries did not demonstrate incremental value under the evaluated conditions. Standardization attenuated the native-scale deficit, identifying implementation sensitivity as a model-selection consideration. Modest discrimination and unstable calibration constrain individual probability interpretation. These results support evaluating added representation complexity against simpler, design-aware benchmarks and assessing calibration alongside discrimination.

## 1. Introduction

Depression is a common mental disorder, and improving early recognition of depression and access to appropriate support remains an important public health and healthcare challenge [1]. The Patient Health Questionnaire-9 (PHQ-9) is widely used to initiate screening, although screening accuracy varies with the selected cutoff and reference diagnostic method [2,3,4]. In the present model-development study, same-session PHQ-9 screen-positive status served as the reference outcome for testing whether interview-derived representations could estimate that status without using questionnaire responses as model inputs. This cross-sectional comparison addresses correspondence with a contemporaneous screening measure; longitudinal predictive validity would require a subsequent outcome assessment. The approximately 30 min fixed-structure interview was a controlled research protocol for eliciting comparable multimodal data. The intended translational use is opportunistic: conversational information would be derived from an interaction already taking place, not from an additional interview intended to replace PHQ-9 screening.

Multimodal representations derived from interviews can provide a traceable source of screening-related information within structured mental health interviews. For healthcare-facing artificial intelligence (AI) model development, discrimination alone does not establish that a more complex representation is useful. Added complexity should improve performance beyond simple, design-aware comparators, and participant-level probabilities should be examined for calibration. A transparent development strategy therefore requires explicit representation comparisons, benchmarks that expose study-design information, and validation of the interview conditions under which multimodal features are informative.

Multimodal AI for depression assessment combines information from speech, facial behavior, and language to characterize depression-related states. Fixed-structure interviews provide a noninvasive, repeatable context in which these signals can be obtained and may therefore offer a research pathway toward supporting depression-screening processes. Earlier speech-based work combined factor analysis (FA) with Gaussian mixture models (GMMs) to classify depression severity, and recent evidence indicates that acoustic depression-related signatures may differ between emotion- and theme-based speech tasks [5,6]. However, heterogeneity in datasets, features, evaluation metrics, and validation methods, a lack of transparency, and performance degradation on external data have been repeatedly noted [7,8,9].

Previous reports based on nested subsets of the same interview corpus addressed dataset construction and exploratory feature–questionnaire correlations in 25 university students [10], segment-level emotion/topic classification using a multifactor FA–GMM framework in 99 participants [11], and deep-fusion classification of a PHQ-9-derived binary outcome in 74 participants [12]. None evaluated the participant-level incremental contrast prespecified here: whether two low-complexity GMM posterior summaries improve performance beyond participant-level means of one-factor FA scores when the analytic sample, cohort covariate, model class, and resampling splits are held constant. The fixed interview comprised happy, sad, anger, and lifestyle phases, which may elicit different acoustic, facial, and linguistic patterns. These phases also introduced a phase-specific question structure [13,14,15]. Interview phase and retained-segment conditions were therefore treated as validation-relevant design dimensions, and the analyses contextualized interview-derived performance in relation to cohort membership and protocol structure.

The central question was whether participant-level means of the factor scores captured the information relevant to estimating PHQ-9 screen-positive status from fixed-structure interviews or whether the GMM summaries of cross-modal distributional heterogeneity across acoustic, visual, and linguistic segments provided incremental value. Participants with similar mean factor scores may nevertheless differ in the proportion of segments assigned to a joint multimodal pattern or in the certainty of those assignments. The GMM summaries were intended to capture these nonlinear distributional differences. Their incremental value depends on whether those differences contain information about the screening outcome beyond the factor means and cohort indicator. If mean levels are sufficient within this representation, summaries that incorporate segment-level distributional information may warrant lower priority. Conversely, incremental value from the GMM summaries would support designs that preserve a cross-modal and within-topic distributional structure. This study operationalized this contrast using one-factor FA scores for each feature representation and GMM-derived posterior summaries in segment-level factor-score space. Topic dependence was not treated as a separate hypothesis in the primary analysis. Instead, retaining the question structure of fixed-structure interviews through the topic phase was a design premise.

Accordingly, the primary objective was to determine whether GMM-derived distributional summaries provided incremental participant-level information for same-session estimation of PHQ-9 screen-positive status beyond one-factor mean representations. A design-only benchmark using only the adult-versus-pediatric cohort indicator contextualized whether the interview-feature models added information beyond study composition. This study also evaluated probability calibration and explored variation across interview phase and retained-segment conditions. Together, these analyses addressed not only whether the evaluated GMM summaries added value but also which dimensions of representation, benchmarking, calibration, and interview design should be made explicit when transparent multimodal AI is developed for depression assessment in healthcare.

## 2. Methods

### 2.1. Study Design, Intended Use, Relation to Previous Reports, and Preregistration

This was a preregistered secondary analysis of a previously reported Japanese-language corpus of fixed-structure interviews. This study evaluated a traceable multimodal AI pipeline that combines acoustic, facial-expression, and linguistic information from counselor-conducted interviews to estimate same-session PHQ-9 screen-positive status. The primary analysis tested whether two participant-level posterior summaries from a prespecified two-component GMM provided incremental value beyond participant-level means of one-factor FA scores under repeated nested cross-validation. A design-only benchmark using the adult-versus-pediatric cohort indicator was included to contextualize performance. All primary model comparisons used the same common analysis set and participant-level split bank.

The 103-participant cohort was the full labeled dataset. It included all 25 participants reported by Kashihara et al. [10], all 99 participants analyzed by Kiuchi et al. [11], and all 74 participants analyzed by Matsumoto et al. [12]. These previously reported samples overlapped and were entirely nested within the current 103-participant cohort. The earlier reports addressed dataset construction and exploratory feature–questionnaire correlations [10], segment-level emotion/topic classification [11], and deep-fusion classification of a PHQ-9-derived binary outcome [12]. The present primary model contrast, participant-level estimand, split bank, and calibration analysis were not reported in the earlier publications. The present study evaluated this contrast using repeated nested cross-validation, average precision (AP) as the primary metric, and an explicit design-only benchmark. The same prespecified participant-level split bank was used across the models compared within the present study and was not reused from the earlier reports. No tables or figures from those reports are reproduced here. Table S24 compares their samples, objectives, representations, models, selected reported results, and validation strategies with those of the present study. Performance values across these studies are contextual summaries, not matched model comparisons.

The analysis plan was preregistered at OSF Registrations on 30 March 2026 (registration: jenz7; anonymous view-only URL: https://osf.io/jenz7/overview?view_only=1a20863a871b4f6497e70bd6aff3280f (accessed on 11 October 2026)). The primary outcome, primary model contrast, primary metric, and  generation rule and base seed for the split bank were fixed before the primary performance estimates were inspected. The prespecified primary analysis compared out-of-sample estimation performance between the four-representation FA and FA+GMM models using the same split bank within the common analysis set. The design-only benchmark, which used only the two-group cohort indicator, was reported as a prespecified reference for interpreting whether the interview-feature models exceeded the study-design information. Prespecified secondary analyses, exploratory analyses, and post hoc analyses were reported separately; they characterized the boundary conditions and implementation sensitivity of the primary finding rather than replacing the primary analysis. The GMM-input scaling sensitivity analysis was post hoc. Additional implementation details are provided in Section S1, and reproducibility details are provided in Section S5.

### 2.2. Participants, Cohort Composition, and PHQ-9 Outcome

The 103 participants in the original study constituted the labeled cohort for this study, and no additional participants were excluded from the common set for the primary analysis. The final analysis sample comprised 83 participants in the adult stratum (15 screen-positive; 18.1%) and 20 participants in the pediatric stratum (9 screen-positive; 45.0%), yielding 24 PHQ-9 screen-positive and 79 screen-negative participants overall. The units of estimation and performance evaluation were participant-level interview sessions; speech segments were treated as internal units for feature extraction and participant-level aggregation. All cross-validation splitting was performed at the participant level. Because this was a secondary analysis, the study size was determined by the full labeled cohort available in the previously collected corpus; no additional participants were recruited for this analysis. No prospective sample-size calculation determined this secondary analysis. The 24 positive outcomes, rather than the number of speech segments or cross-validation repeats, constrained the precision of the participant-level performance estimates. Each outer-validation fold contained only approximately four or five positive participants; repeated splitting assessed split-related variability without increasing the number of independent participants.

A post hoc precision analysis quantified uncertainty in the primary repeat-mean model contrast and examined evaluation sample sizes under explicit assumptions about outcome prevalence and the joint distribution of predictions. The calculation targeted a range of interval widths, following the principle that precision requirements should be specific to the estimand and evaluation setting [16]. The methods and sample-size sensitivity results are reported in Section S10.

The source cohort comprised university students, adult patients, and pediatric patients. The initial 25-participant university-student subset completed research interviews in a university experimental setting.

The primary outcome was PHQ-9 screen-positive status, defined as a pre-interview PHQ-9 total score of 10 or higher. The PHQ-9 was completed before the interview in the same session, and interview-derived features were generated after the complete interview was available. The outcome therefore represented same-session correspondence between questionnaire status and information from the subsequent interview rather than prospective risk prediction. The Japanese version of the Patient Health Questionnaire-9 (J-PHQ-9), validated by Muramatsu et al. [17], was administered in both the adult and pediatric strata. The pediatric stratum comprised 20 participants aged 11–17 years, with a median age of 14.5 years. Participants primarily completed the questionnaire themselves. Researchers or research staff checked participants’ understanding of the items and provided clarification and completion support as needed. The scored responses were provided by participants rather than by proxy respondents.

The threshold of 10 was selected based on evidence for the original PHQ-9 and the J-PHQ-9 in adult primary-care populations [3,17,18]. To preserve the prespecified outcome, the same J-PHQ-9 and threshold were applied in both strata. Because criterion validity for the administered form and cutoff had not been established in pediatric participants, their screen-positive status was treated as a study-specific operational label. A criterion validity study of the PHQ-9 among adolescents aged 13–17 years identified an optimal threshold of 11 [19], whereas a Japanese general-population study of the PHQ-A reported score distributions without establishing a cutoff [20].

As a post hoc outcome-threshold sensitivity analysis, the threshold of 10 was retained for the adult stratum, and the threshold of 11 reported for adolescents was applied to the pediatric stratum. We refitted the design-only benchmark, the three- and four-representation FA and native-scale FA+GMM models, and the four-representation standardized-input FA+GMM model under the revised labels. The original 25-repeat, five-outer-fold, four-inner-fold split bank was retained without restratification. Preprocessing, FA, GMM, and penalty selection were repeated within the training splits using the original settings. The prespecified threshold-10 analysis remained the primary analysis. Section S11 provides the sensitivity analysis methods and results, including the single-retry procedure for ridge convergence failures.

In the primary analysis, a two-group cohort indicator was entered as a fixed covariate: university students and adult patients were grouped into the adult stratum, and pediatric patients were grouped into the pediatric stratum. This study-specific design variable may capture differences in age, recruitment population, patient-versus-student composition, and screen-positive prevalence (18.1% in the adult stratum and 45.0% in the pediatric stratum). Therefore, the main quantity reported in this study is the incremental value of the GMM-derived posterior summaries conditional on the two-group cohort indicator within the same common analysis set and under identical splitting conditions, not a performance estimate within age strata or recruitment populations. Including this indicator does not eliminate heterogeneity within either stratum or establish that the outcome has identical measurement properties across strata. The adult-only sensitivity analysis retained the university-student versus adult-patient distinction to examine the comparison without the pediatric participants (Section S6a).

### 2.3. Multimodal Interview Signals and Traceable Participant-Level Representations

The source data were collected through videoconference interviews lasting approximately 30 min and conducted in Japanese by qualified psychological counselors. Speech intelligibility was checked during the interviews and by replaying the saved audio recordings. Interviewers were blinded to participants’ PHQ-9 total scores and screen-positive status. The fixed protocol elicited participant speech across happy, sad, anger, and lifestyle topic phases and captured acoustic, facial, and linguistic information. These phases were retained as explicit design contexts in auxiliary analyses rather than treated as interchangeable samples of free conversation.

In this manuscript, multimodal AI refers to the complete machine learning pipeline that extracted and integrated acoustic, facial-expression, and linguistic representations to estimate the screening-related outcome. The use of gpt-4o-transcribe was one preprocessing component rather than the target system evaluated.

The primary analysis used four feature representations: acoustic features from openSMILE, self-supervised speech representations from HuBERT, visual facial-expression features from PyFeat, and text embeddings derived from participant speech [21,22,23]. The canonical transcripts were generated from participant speech using gpt-4o-transcribe and were used without manual correction. No accuracy assessment against reference transcripts was available, so word error rates were not quantified before embedding. Structural checks of transcript availability, alignment, and segment availability are detailed in Section S5; the corresponding limitation for text-derived features is addressed in Section 4.5 and in the Use of Artificial Intelligence statement. The internal unit for feature extraction and alignment was the speech segment defined by participant identifier, start time, and end time; in the primary analysis, the four feature representations were aligned on the same speech segments. For the text representation, the frozen sentence encoder intfloat/multilingual-e5-base was used. The dimensionality of feature blocks, retained PyFeat columns, valid-frame rates, alignment rules, and quality control of feature availability at the speech-segment level are presented in Section S5 and Table S19.

Within each inner- and outer-training split, each of the four feature representations was separately reduced to segment-level factor scores using unrotated one-factor FA with minimum residual (MINRES) extraction [24]. The number of factors was fixed at one to improve traceability and stability while limiting overfitting given the small sample of 103 participants, including 24 screen-positive participants. This fixed capacity was not intended to explain all variability in each feature block. Table S1 reports the available loading and score-scale diagnostics, and the two-factor-capacity sensitivity analysis evaluates one prespecified alternative (Table S5). Section S9 summarizes the stored FA diagnostics across outer-training folds; these summaries do not establish featurewise loading stability or explained variance. Factor scores for openSMILE, HuBERT, and PyFeat were aggregated at the participant level as segment-length-weighted means, and the text factor was aggregated as a token-weighted mean.

The GMM-derived posterior summaries were created by fitting a two-component GMM to the segment-level factor-score space of the four feature representations. The participant-level features were the segment-length-weighted mean posterior probability of membership in ordered component 2 (a soft component-2 occupancy summary) and the segment-length-weighted mean difference between the largest and second-largest posterior probabilities (a posterior assignment-margin summary). Ordered component 2 was defined within each outer-training fold as the component with the larger sum of sign-aligned factor-score means. Neither summary represents a regression residual, an explicit residual after FA, or a conventional measure of distributional skewness. The choice of two components does not represent a clinical subtype hypothesis; it is a low-complexity specification for testing the incremental value of adding minimal distributional summaries to the mean factor scores [25].

Consistent with the preregistered specification, the four factor-score dimensions were entered into each GMM on their fold-specific native scales; no additional cross-representation z-standardization was applied before GMM fitting. Participant-level predictors were subsequently standardized within each training split before ridge logistic regression.

The primary comparison includes three models: the design-only model, which used only the two-group cohort indicator and was not treated as a clinical model; the FA model, which used the cohort indicator and one participant-level mean FA score from each of the openSMILE, HuBERT, PyFeat, and text representations; and the FA+GMM model, which added the two GMM-derived posterior summaries. The numbers of input variables were 1, 5, and 7, respectively.

In this study, “transparent” refers operationally to the prespecified low-dimensional predictor structure and explicit model comparisons: each participant-level input can be traced to the cohort indicator, a factor mean from a named feature representation, or one of the two mathematically defined GMM posterior summaries. This usage does not imply that individual high-dimensional source features or model outputs have a direct clinical interpretation.

### 2.4. Internal Validation, Calibration, and Evaluation Metrics

We used ridge logistic regression as the final estimation model, with regularization strength selected by inner cross-validation [26]. Performance was estimated using participant-level repeated nested cross-validation. We performed 25 repeats of stratified 5-fold outer cross-validation and used stratified 4-fold inner cross-validation within the outer-training participants. The stratification rules and acceptance criteria for GMM fits are described in Section S1. Preprocessing and model fitting—including zero-variance-column removal, FA, sign fixing, GMM fitting, standardization, and regularization-strength selection—were performed using the relevant training data only. In each inner split, representations were estimated using the inner-training participants and applied to the inner-validation participants. After penalty selection, the representations and final regression model were refitted using all outer-training participants and applied to the held-out outer fold. Participant-level aggregation was then performed separately for the training and validation sets using the fold-specific fitted representations. This design was intended to reduce the optimistic bias that can arise when model selection and performance evaluation use the same data [27].

The primary evaluation metric was the average precision (AP) difference, calculated as the FA+GMM model minus the FA model. AP was prioritized because the outcome was imbalanced and the metric summarizes precision–recall performance relative to the observed screen-positive prevalence. Because AP depends on the positive rate of the evaluation set, the screen-positive rate of 24/103 = 0.233 was interpreted as the no-skill AP baseline in the common set for the primary analysis [28]. The area under the receiver operating characteristic curve (ROC-AUC) assessed ranking discrimination, the Brier score assessed overall probabilistic accuracy, and calibration intercepts and slopes assessed the calibration of participant-level probability estimates. The design-only model was included to assess the extent to which differences between the adult and pediatric strata, rather than interview-derived information, contributed to apparent performance. No operational probability threshold was prespecified; threshold-dependent measures were therefore outside the analysis plan and were not used to draw operational conclusions. This study did not perform formal hypothesis tests or make significance claims based on p-values; instead, it describes the direction and magnitude of paired metric differences under the same sample and splitting conditions.

Calibration intercepts and slopes were used to assess the calibration of participant-level probability estimates. Within each outer repeat, the logistic recalibration coefficients for each validation fold were estimated from predictions in the remaining outer folds and applied to that held-out fold. This procedure reused the existing stratified outer-fold assignments; it did not create a new stratified partition at the recalibration stage. The intercept and slope reported after recalibration assessed the pooled cross-fitted recalibrated predictions and were not the fold-specific coefficients themselves. Calibration intercepts were estimated jointly with slopes in a logistic regression on the logit of the estimated probability; they were not intercept-only estimates with the slope fixed at one. Calibration coefficients before and after recalibration were summarized as means and standard deviations across evaluable repeats. In a post hoc analysis, conditional 95% confidence intervals for these means were obtained from 2000 participant-level bootstrap samples. Each participant’s predictions across all repeats and models were resampled together, and the assessment intercept and slope were refitted within each repeat before averaging. Predictions and fitted recalibration transformations were held fixed; these intervals did not include uncertainty from retraining the prediction models or recalibrators. Section S1 provides the resampling details. Because fold-specific transformations can alter rankings when validation-fold predictions are pooled, the recalibrated AP and ROC-AUC were treated as auxiliary descriptive metrics rather than as conventional ranking measures. This recalibration was a postprocessing diagnostic on the shared outer-CV predictions, rather than a fully nested evaluation in which the calibrator and all models supplying its inputs were fitted exclusively within each outer-training set. As an auxiliary uncertainty assessment, each participant’s out-of-sample probability estimates were averaged across evaluable repeats to obtain one participant-level mean probability, and 95% conditional bootstrap intervals were calculated using an outcome-stratified participant-level percentile bootstrap with 2000 resamples. Paired intervals were calculated for the FA+GMM model minus the FA model, the FA model minus the design-only model, and the FA+GMM model minus the design-only model, using the repeats evaluable for both models in each comparison. The outcome-stratified resampling retained the observed numbers of screen-positive and screen-negative participants. These intervals are conditional on the prespecified split bank and the already-generated out-of-sample estimated probabilities. The bootstrap did not refit the complete pipeline and therefore did not propagate uncertainty from alternative data splits, split-specific preprocessing and representation learning, hyperparameter selection, or model fitting. Accordingly, these intervals are not full-pipeline uncertainty intervals, formal hypothesis tests, or evidence of external validity.

For the post hoc precision analysis, we additionally calculated conditional 95% intervals for the mean paired AP, ROC-AUC, and Brier differences across the 24 common repeats. We used 2000 ordinary participant bootstrap samples, retaining each participant’s full prediction vector across models and repeats, and averaged the paired metric differences calculated within each repeat. This preserved the primary estimand while allowing the positive count to vary. For sample-size sensitivity, we simulated independent evaluation samples from the empirical joint distribution of outcomes and prediction vectors, varying prevalence and total sample size. Target widths and all simulation settings were fixed before these additional calculations. Predictions and fitted recalibration transformations were held fixed; only the calibration assessment regressions were refitted. Consequently, the projected sample sizes concern evaluation precision under these distributional assumptions and do not determine the sample size required to develop the complete FA–GMM pipeline.

For the primary threshold-10 analysis, all 25 planned repeats were attempted. In accordance with the analysis plan, a repeat contributed to a model summary only when all participant-level predictions for that repeat were estimated; not_estimated was retained as a method failure rather than treated as a prediction. One outer-fold fit of the FA+GMM model failed because an inner-fold logistic regression remained nonconvergent after the single retry specified in the analysis plan, with the iteration limit increased from 10,000 to 20,000 (repeat 8, outer fold 2; Table S18). Consequently, the design-only model and FA model summaries used 25 evaluable repeats, the FA+GMM model summary used 24, and the paired primary comparison and its bootstrap difference were based on the 24 common evaluable repeats. The benchmark comparisons used 25 common repeats for the FA model and 24 common repeats for the FA+GMM model. Model-specific bootstrap summaries used all evaluable repeats for each model.

### 2.5. Auxiliary Analyses, Reproducibility, and Ethics

Prespecified secondary and sensitivity analyses evaluated specifications with three feature representations, phase-aware specifications, alternative factor and GMM complexity, and an alternative text representation. In the cap2 sensitivity, each modality had a fixed two-factor capacity, with the second factor set to zero when only one factor was estimable. The three-component-GMM sensitivity summarized the ordered second- and third-component posterior probabilities and the posterior assignment margin; because only GMM complexity changed, it reused the primary four-representation FA model as its comparator. These fold-specific representations were fitted within the inner- and outer-training splits and used the primary participant split bank. Exploratory auxiliary analyses evaluated interview-phase-specific fixed-summary performance, length-stability sensitivity, descriptive overlap between GMM summaries and mean FA scores, sensitivity to residualizing fixed GMM summaries, adult-only cohort-composition sensitivity, and GMM-input scaling sensitivity. These analyses did not replace the primary analysis; instead, they examined potential boundary conditions of the main results, sensitivity to representational choices, and dependence on the fixed-interview structure.

Two exploratory nested-cross-validation diagnostics used participant summaries that had been precomputed from the full source dataset; neither diagnostic re-estimated FA or GMM within the cross-validation splits. The phase-specific diagnostic compared three-representation mean FA summaries with and without phase-specific GMM summaries using the 25-repeat topic split bank. The overall fixed-summary diagnostic compared four-representation mean FA summaries, the corresponding raw GMM summaries, and GMM summaries residualized on the four mean FA summaries within each inner- or outer-training fold, using the 25-repeat primary split bank. Ridge logistic-regression fitting and penalty selection were repeated within the nested splits. Because representation construction was fixed before cross-validation, these diagnostics were treated as exploratory downstream-model comparisons and not as full-pipeline internal-validation estimates. Their held-out participants contributed to representation estimation; the downstream CV therefore does not remove optimism introduced at that stage.

The post hoc GMM-input scaling sensitivity analysis changed only the scaling of the four segment-level FA scores supplied to the GMM. Within each inner and outer fit, means and population standard deviations were estimated from the participant-balanced GMM training segments and then applied to the training segments and the corresponding validation or test segments. The participant split bank, FA representations, random-seed bank, GMM settings, downstream model, and evaluation procedures were otherwise held fixed. The primary scaling-sensitivity contrast was the standardized-input FA+GMM model minus the native-scale FA+GMM model for raw AP; the standardized-input FA+GMM model minus the FA model evaluated incremental value under the alternative scaling specification. Raw ROC-AUC and Brier scores, together with recalibrated metrics, were supporting comparisons. Full methods and validation checks are provided in Section S6b.

The primary analysis and the prespecified secondary and other auxiliary analyses were performed using Python 3.12.2 with SciPy 1.15.1, scikit-learn 1.6.1, factor-analyzer, and transformers 4.57.6. statsmodels was used for mixed-effects analyses. The post hoc GMM-input scaling analysis, fixed-summary diagnostics, and pediatric outcome-threshold sensitivity analysis used an isolated validation environment running Python 3.11.15 with NumPy 2.4.4, SciPy 1.15.1, and scikit-learn 1.6.1. During revision, OpenAI Codex assisted with developing and checking code for additional conditional confidence intervals and evaluation-precision analyses, as well as with interpreting these analyses. The graphical abstract was revised using OpenAI image generation through Codex, with text and numerical values checked against this manuscript. The policy for sharing data and code is described in the “Data Availability” statement, and the scope of artificial intelligence (AI) use is described in “Use of Artificial Intelligence.” This study was approved by the Ethics Committee of Tokushima University Hospital (Approval Number: 3650; Approval Date: 18 December 2023). Written informed consent was obtained from the adult participants; for minors, oral assent was obtained from the participants, and written consent was obtained from their guardians.

## 3. Results

### 3.1. Participants and Analyzable Data

The labeled cohort included 103 participants, with 24 screen-positive and 79 screen-negative participants. Table 1 summarizes the adult and pediatric strata, including age, sex, PHQ-9 score distribution, and screen-positive frequency. The common set for the primary analysis was defined as the entire labeled cohort of 103 participants, with no additional exclusions. In the prespecified phase-aware secondary analysis, participants with defined participant-level summaries for all four phases—happy, sad, anger, and lifestyle—comprised the secondary-analysis common set PA*. PA* included 102 participants, after excluding one participant from the primary-analysis common set. Topic-specific mixed-effects analysis required at least two analyzable phases, a criterion met by all 103 participants; among them, 102 had all four phases analyzable and the remaining participant had two analyzable phases.

Screen-positive status was more frequent in the pediatric stratum (9/20, 45.0%) than in the adult stratum (15/83, 18.1%). When the adult stratum was divided into university students and adult patients, the corresponding proportions were 6/34 (17.6%) and 9/49 (18.4%), respectively (Table S2).

Under the post hoc outcome-threshold definition that retained the threshold of 10 in the adult stratum and applied the threshold of 11 reported for adolescents in the pediatric stratum, two pediatric participants with PHQ-9 scores of 10 were reclassified as screen-negative. The screen-positive count in the pediatric stratum consequently decreased to 7/20 (35.0%), and the overall count decreased to 22/103 (21.4%). Model-performance results under this definition are reported in Section 3.8.

Thus, the adult-versus-pediatric stratum indicator in the primary-analysis common set was a design variable that captured differences in age group, recruitment population, patient-versus-student composition, and screen-positive prevalence.

For the descriptive availability analysis, the inputs available for outcome-blind factor estimation comprised 15,088 openSMILE, 15,088 HuBERT, 14,957 PyFeat, and 15,082 text segments. For performance estimation, all preprocessing, representation fitting, GMM fitting, and participant-level aggregation steps were performed anew for each split. Because of the differences in availability across modalities, the common set of segments with all four feature representations contained 14,952 segments. The participant-level median number of such segments was 155, the median total duration of these segments was 747,619 ms, and the median total number of text tokens was 1677. When stratified by screen-positive status and cohort stratum, no large imbalance was observed in the number of complete four-representation segments or in the PyFeat exact-join usable rate (Table S19).

The results are presented below in the following order: prespecified primary analysis, comparisons with the design-only model, recalibration, prespecified secondary analyses, and exploratory auxiliary analyses. Detailed numerical tables for sensitivity analyses and exploratory comparisons are provided in the appendices.

### 3.2. Internal Validation of PHQ-9 Screen-Positive Estimation

Using identical participant splits within the common analysis set of 103 participants, the design-only model had mean AP, ROC-AUC, and Brier scores of 0.332, 0.612, and 0.169, respectively, across 25 evaluable repeats. These were the highest AP and ROC-AUC and the lowest Brier score among the three models reported in the main text (Table 2). The FA model had corresponding mean metric values of 0.302, 0.576, and 0.177 across 25 repeats. The FA+GMM model had corresponding values of 0.282, 0.546, and 0.179 across 24 repeats because one planned repeat was not evaluable. The screen-positive rate in the common set was 24/103 = 0.233. Relative to this no-skill AP baseline, the AP excess was +0.069 for the FA model and +0.049 for the FA+GMM model. The model-specific AP estimates based on participant-mean probabilities were 0.327 (95% conditional bootstrap interval, 0.255–0.501) for FA and 0.279 (0.229–0.397) for FA+GMM. These auxiliary estimates evaluate the aggregation in Table 2, Panel B, rather than mean performance across repeats.

For the prespecified primary contrast (the FA+GMM model minus the FA model), the mean paired differences across the 24 common evaluable repeats were ΔAP −0.020, ΔROC-AUC −0.031, and ΔBrier +0.0016 (Table 3). The post hoc participant-bootstrap 95% conditional intervals for these repeat-mean differences were −0.046 to +0.001, −0.053 to −0.008, and +0.00043 to +0.00294, respectively. The interval for the primary AP contrast included zero.

Under the fixed empirical distribution with a positive proportion of 24/103, the first tested evaluation sample size meeting an approximate 95% interval full-width target of 0.02 for the AP difference was 800 (approximately 186 positive participants). A slope-width target of 0.40 for either recalibrated model was first met at 6400 (approximately 1491 positive participants). These conditional planning scenarios and alternative targets are reported in Tables S26 and S27.

In the auxiliary comparison based on each participant’s mean out-of-sample estimated probability across these repeats, the paired conditional estimates (95% bootstrap intervals) were −0.048 (−0.147 to −0.008) for ΔAP, −0.046 (−0.083 to −0.011) for ΔROC-AUC, and +0.00153 (+0.00041 to +0.00281) for ΔBrier. The mean differences across repeats and conditional estimates use different summary bases and therefore are not expected to be identical. These intervals condition on the generated out-of-sample predictions and split bank and do not propagate full-pipeline uncertainty.

Across repeats, differences predominantly favored the FA model. For the FA+GMM model minus the FA model, repeat-level ΔAP had a median of −0.018, an interquartile range (IQR) of −0.037 to −0.004, and a range of −0.091 to +0.040. ΔAP was positive in 3 of 24 common evaluable repeats. ΔROC-AUC had a median of −0.036, an IQR of −0.048 to −0.018, and a range of −0.080 to +0.046. ΔROC-AUC was positive in 3 of 24 repeats. ΔBrier had a median of +0.00100, an IQR of +0.00007 to +0.00170, and a range of −0.00044 to +0.01292; the FA+GMM model had a lower Brier score in 3 of 24 repeats. These repeats represent variability in internal validation induced by re-splitting the same participants; they are not independent external validations.

For implementation context, the post hoc standardized-input FA+GMM model had a mean AP of 0.300 across 25 evaluable repeats, compared with 0.282 for the primary native-scale FA+GMM model and 0.302 for the FA model. Because the standardized model was not prespecified, it is reported separately as a sensitivity analysis in Tables S22 and S23 and does not replace the primary contrast.

**Figure 1:**
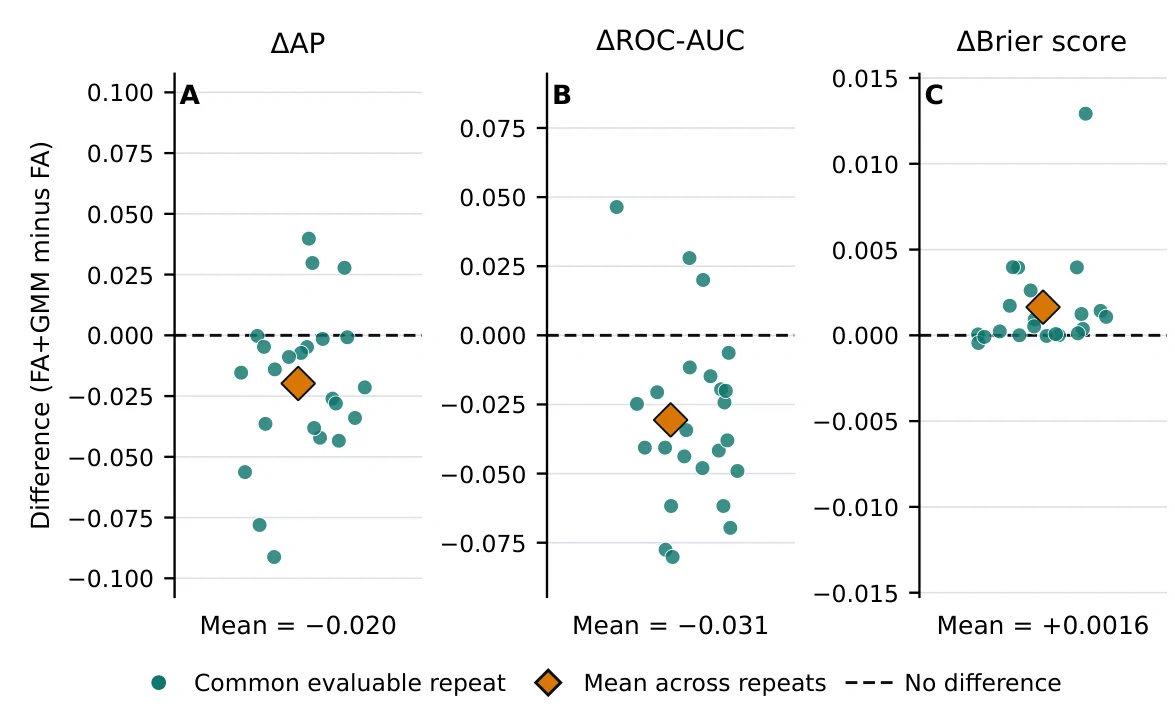
Repeat-level paired differences in the primary comparison of the four-representation FA+GMM and FA models. (**A**) Average precision (AP) differences; (**B**) area under the receiver operating characteristic curve (ROC-AUC) differences; (**C**) Brier score differences. Each circle represents one of the 24 common evaluable repeats, diamonds denote means across repeats, and the dashed horizontal line denotes no difference. Differences are FA+GMM minus FA. Thus, positive values favor FA+GMM for AP and ROC-AUC, whereas negative values favor FA+GMM for Brier score. The entire repeat 8 was excluded under the prespecified method-failure rule because an inner-fold logistic regression remained nonconvergent for FA+GMM in outer fold 2 after the specified single retry. Abbreviations: AP, average precision; FA, factor analysis; GMM, Gaussian mixture model; ROC-AUC, area under the receiver operating characteristic curve.

Figure 1 displays the primary incremental comparison at the repeat level. Across the 24 common evaluable repeats, the mean FA+GMM-minus-FA differences were ΔAP −0.020, ΔROC-AUC −0.031, and ΔBrier +0.0016. Most repeat-level differences favored the FA model: positive differences favored FA+GMM for AP and ROC-AUC, whereas negative differences favored FA+GMM for Brier score. Participant-mean estimated probability distributions are shown on both the full probability scale and a clearly identified zoomed scale in Figure S6.

### 3.3. Study-Design Benchmark and Probability Calibration

As shown in Table 2 and Table 3, mean point estimates across repeats favored the design-only model, whereas all paired conditional intervals for comparisons with this model included zero.

In the adult-only cohort-composition sensitivity analysis, the adult-only analysis set A* comprised 83 participants in the adult stratum (15 screen-positive and 68 screen-negative participants). The adult-source design-only model and interview-feature models were compared using the within-adult source variable indicating university student versus adult patient. Across 25 repeats, the adult-source design-only model had mean AP, ROC-AUC, and Brier values of 0.164, 0.463, and 0.149; the adult-source FA model had corresponding values of 0.212, 0.509, and 0.148; and the adult-source FA+GMM model had corresponding values of 0.197, 0.483, and 0.149. Paired differences were ΔAP +0.051, ΔROC-AUC +0.037, and ΔBrier −0.00002 for the adult-source FA model minus the adult-source design-only model and ΔAP −0.016, ΔROC-AUC −0.026, and ΔBrier +0.00015 for the adult-source FA+GMM model minus the adult-source FA model (Tables S20 and S21).

Logistic recalibration with leave-one-outer-fold-out cross-fitting yielded AP, ROC-AUC, and Brier values of 0.323, 0.611, and 0.168 for the design-only model; 0.279, 0.540, and 0.182 for the FA model; and 0.263, 0.515, and 0.185 for the FA+GMM model, respectively (Table 4). The corresponding calibration slopes of the cross-fitted recalibrated probabilities were 0.970, 0.104, and 0.093, respectively. Before recalibration, mean slopes were 1.412, 1.304, and 0.372, respectively. For FA and FA+GMM, the corresponding between-repeat SDs were 1.924 and 2.160 before recalibration and 0.941 and 0.680 afterward, indicating substantial split-related variability. Conditional 95% confidence intervals for the mean post-recalibration slopes were −1.138 to 1.477 for FA and −1.238 to 1.581 for FA+GMM, indicating limited precision. Recalibration increased mean Brier scores from 0.177 to 0.182 for FA and from 0.179 to 0.185 for FA+GMM. Because fold-specific recalibration can alter rankings across validation observations, the recalibrated AP and ROC-AUC values are reference values rather than ranking metrics obtained by applying a single monotone transformation.

The low calibration slopes of the cross-fitted recalibrated probabilities for the interview-feature models (0.104 for the FA model and 0.093 for the FA+GMM model), together with recalibrated Brier scores that did not improve on the design-only model, did not support interpreting their outputs as calibrated individual-level screening probabilities. No operational probability threshold was derived.

**Figure 2:**
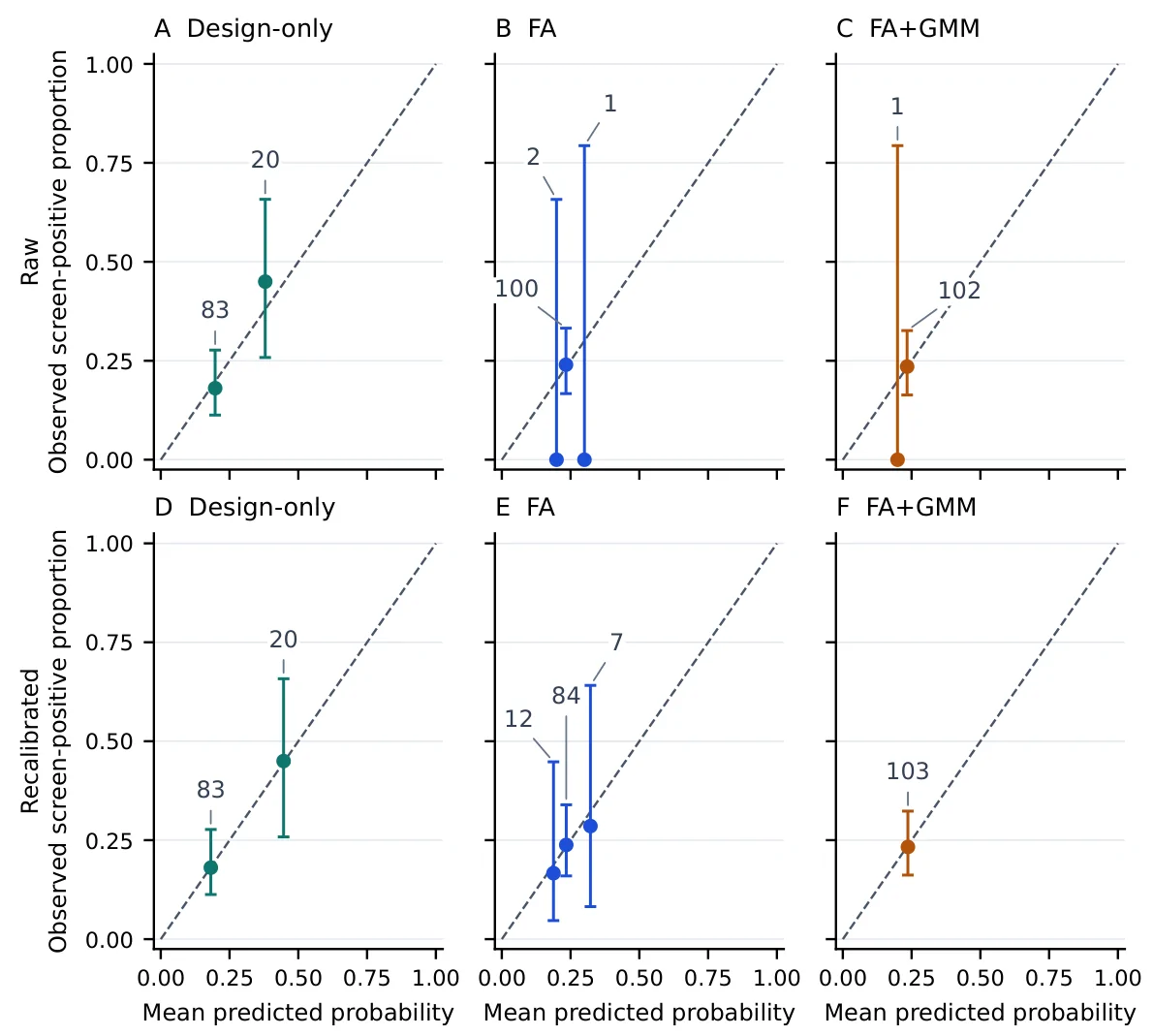
Descriptive calibration of participant-averaged probabilities. (**A**) Raw design-only probabilities; (**B**) raw FA probabilities; (**C**) raw FA+GMM probabilities; (**D**) recalibrated design-only probabilities; (**E**) recalibrated FA probabilities; (**F**) recalibrated FA+GMM probabilities. Recalibrated probabilities were obtained using the existing leave-one-outer-fold-out recalibration. All panels include the same 103 participants (24 screen-positive), using the 24 common evaluable repeats; Repeat 8 is excluded because of the FA+GMM convergence failure. Each participant contributes once per panel, after averaging probabilities across repeats. Points show the mean predicted probability and observed screen-positive proportion in fixed-width probability bins of 0.1; empty bins are omitted, identical probabilities remain together, and numbers indicate participants per bin; short leader lines connect count labels to the upper ends of the corresponding intervals. Vertical bars are descriptive 95% Wilson binomial intervals for the observed proportions, conditional on the predictions and binning. They are not intervals for calibration coefficients and do not account for dependence from overlapping training samples or uncertainty from model development. The dashed line indicates perfect calibration. Recalibrated panels depict postprocessing of shared cross-validation predictions, not fully nested validation of the complete recalibration pipeline. FA, factor analysis; GMM, Gaussian mixture model.

Figure 2 displays binned calibration for participant-averaged probabilities across the 24 common evaluable repeats. Most interview-feature probabilities occupied a narrow range, leaving several bins empty or sparsely populated. This descriptive aggregation complements the repeat-level coefficients in Table 4; it does not replace their assessment of split-related variability.

### 3.4. Summary of Prespecified Secondary Analyses

The prespecified three-representation analysis excluded transcript-derived text and used openSMILE, HuBERT, and PyFeat. In this analysis, S* denoted the same 103-participant common set used for the primary comparison, and the primary split bank was retained. The FA model had raw AP, ROC-AUC, and Brier values of 0.336, 0.618, and 0.175, respectively, whereas the FA+GMM model had corresponding values of 0.299, 0.581, and 0.178. The four-representation rows using the current text representation in Table S5 repeat the primary models for provenance and therefore match Table 2 exactly: AP was 0.302 for the primary FA model and 0.282 for the primary FA+GMM model. Table S14 distinguishes these models from the T* text-representation sensitivity models and documents the analysis set, split bank, representation-fitting strategy, prediction-completeness rule, evaluable repeats, and run identifiers.

In the alternative-complexity analyses, all models contributed 25 evaluable repeats. With a fixed two-factor capacity per modality (cap2), the FA model had mean raw AP, ROC-AUC, and Brier values of 0.303, 0.558, and 0.177, respectively, compared with 0.287, 0.549, and 0.178 for FA+GMM. In the three-component-GMM sensitivity, the reused primary FA comparator had corresponding values of 0.302, 0.576, and 0.177, whereas the FA+GMM model had values of 0.268, 0.521, and 0.179. The recalibrated point estimates had the same direction in both sensitivity analyses (Table S5 and Figure S1).

In the prespecified phase-aware secondary analysis using PA*, which included 102 participants for whom all four phases were analyzable (24 screen-positive and 78 screen-negative participants), the paired mean differences across repeats for phase-aware FA+GMM minus phase-aware FA were ΔAP −0.008, ΔROC-AUC −0.014, and ΔBrier −0.0001 for the raw metrics, and ΔAP −0.009, ΔROC-AUC −0.012, and ΔBrier +0.0019 after recalibration (Table S6).

### 3.5. Exploratory Interview Phase and Length-Stability Analyses

In the exploratory fixed-summary comparison, lifestyle had the highest mean raw AP and ROC-AUC across repeats for both the three-representation FA model (0.335 and 0.652) and the corresponding FA+GMM model (0.326 and 0.628; Table 5). Adding the fixed GMM summaries did not consistently improve phase-specific point estimates: ΔAP ranged from −0.015 to +0.005, and ΔROC-AUC was negative in all four phases (−0.027 to −0.012). Because the FA and GMM summaries were precomputed before cross-validation, this diagnostic estimated downstream-model performance for the fixed summaries rather than full-pipeline internal-validation performance by phase.

In the length-stability sensitivity analysis, the first 16, 32, and 48 segments corresponded empirically to approximately 1, 2, and 3 min of participant speech; the same-phase full comparator represented approximately 3–5 min for the emotional phases and 2–4 min for lifestyle (Table S10). The performance gaps varied nonmonotonically by phase and retained their segment count (Table S9). For happy, the AP and ROC-AUC deficits relative to the full comparator narrowed from −0.120 and −0.147 with 16 retained segments to −0.020 and −0.023 with 48 retained segments. For lifestyle, the AP and ROC-AUC gaps with 32 retained segments were +0.003 and +0.050, respectively, whereas the raw Brier score was worse by +0.095. The exploratory analysis did not identify a minimum retained-segment condition associated with stable performance. Cell sizes varied; for lifestyle with 48 retained segments, the paired analysis sets contained a mean of only 3.9 screen-positive participants per repeat.

### 3.6. Summary of Exploratory Auxiliary Analyses

Across the text-representation, distributional-extension, and fixed-summary residualization analyses, GMM-derived summaries did not show consistent improvement beyond the corresponding mean FA scores. In exploratory descriptive diagnostics based on precomputed fixed summaries, the four participant-level mean FA scores explained 76.3% and 66.4% of the variance in the soft component-2 occupancy summary and posterior assignment-margin summary, respectively. In the fixed-summary cross-validation diagnostic, fold-wise residualization improved the GMM model relative to its raw-summary counterpart (AP, 0.298 versus 0.284; ROC-AUC, 0.561 versus 0.553), but its point estimates remained less favorable than those of the fixed-summary FA model (AP, 0.309; ROC-AUC, 0.593). The paired residualized-GMM-minus-FA differences were −0.011 for AP, −0.032 for ROC-AUC, and +0.0015 for the Brier score. Because the underlying FA and GMM summaries were fixed, this comparison was an exploratory downstream-model diagnostic rather than a full-pipeline internal-validation estimate. The planned mixed-effects analyses were not estimable. Full results and analysis provenance are provided in Tables S4, S7, S8, and S11–S14.

### 3.7. Post Hoc GMM-Input Scaling Sensitivity

The post hoc input-scaling sensitivity analysis was motivated by the median outer-fold factor-score standard deviation of 35.658 for openSMILE, compared with 0.975 for HuBERT, 0.944 for PyFeat, and 1.030 for text (Table S1). The preregistered native-scale FA+GMM model had a mean raw AP of 0.282 across 24 evaluable repeats, whereas the standardized-input model had a mean raw AP of 0.300 across 25 repeats; the FA model had a mean raw AP of 0.302 across 25 repeats. The standardized-input model had a mean raw ROC-AUC of 0.565 and a Brier score of 0.1771, compared with 0.576 and 0.1772, respectively, for the FA model. Across 25 paired repeats, the standardized-input FA+GMM model minus the FA model had mean differences of −0.0014 for AP, −0.0103 for ROC-AUC, and −0.00010 for the Brier score. For the separate estimand based on participant-mean probabilities, conditional intervals included zero for all three contrasts. Thus, training-fold standardization substantially attenuated the performance deficit of the primary native-scale GMM specification, but the standardized GMM summaries still showed no clear incremental value beyond the mean FA representations. Full model-specific and paired results are provided in Tables S22 and S23.

### 3.8. Post Hoc Pediatric Outcome-Threshold Sensitivity

With the adult threshold retained at 10 and the pediatric threshold changed to 11, the no-skill AP baseline was 22/103 = 0.214. Mean raw AP was 0.234, 0.220, and 0.232 for the four-representation FA, native-scale FA+GMM, and standardized-input FA+GMM models, respectively, across 25 evaluable repeats. Their mean paired AP differences were −0.0146 for native-scale FA+GMM minus FA, −0.0023 for standardized-input FA+GMM minus FA, and +0.0123 for standardized-input minus native-scale FA+GMM. The design-only benchmark had a mean raw AP of 0.217, below that of FA under this outcome definition. In the three-representation analysis, mean raw AP was 0.255 for FA across 25 repeats and 0.238 for FA+GMM across 24 repeats; the mean paired difference was −0.0182 over the 24 common repeats. One inner-fold logistic regression remained nonconvergent after the single retry specified in the analysis plan with a maximum of 20,000 iterations, excluding repeat 20 from the three-representation FA+GMM summary. The other five models had no failed outer-fold units (Table S28).

For the separate estimand based on participant-mean probabilities across repeats, the native-scale FA+GMM-minus-FA AP difference was −0.0335 (conditional 95% interval, −0.0944 to −0.0088); the standardized-input FA+GMM-minus-FA difference was +0.0041 (−0.0276 to +0.0428). These intervals apply to differences calculated after averaging each participant’s probabilities, not to the repeat-mean differences above. Table S29 reports both estimands separately.

## 4. Discussion

### 4.1. Principal Findings

This study examined whether two GMM-derived posterior summaries improved estimation of same-session PHQ-9 screen-positive status at the participant level beyond participant-level mean FA scores in multimodal interviews conducted by counselors. The preregistered native-scale FA+GMM implementation had less favorable mean AP, ROC-AUC, and Brier estimates than FA. In a post hoc sensitivity analysis, training-fold standardization increased mean raw AP from 0.282 to 0.300, close to the FA model’s 0.302, and therefore substantially attenuated this deficit. However, the standardized GMM summaries still showed no clear incremental value beyond the mean FA representations. Across the prespecified secondary analyses and the exploratory and post hoc sensitivity analyses reported here, the GMM-derived summaries did not show consistent incremental benefit over their corresponding FA models. Neither increasing the factor capacity to two per modality nor increasing the GMM specification to three components reversed the direction of the within-family point-estimate comparisons.

This pattern separates implementation sensitivity from incremental value. The native-scale performance deficit was partly sensitive to input scaling, whereas standardization did not reverse the incremental-value conclusion. Refitting under the alternative pediatric threshold of 11 likewise did not demonstrate incremental value from the evaluated GMM summaries. This result does not establish either the general inferiority of GMMs or equivalence between representations. It shows that adding these two GMM summaries did not demonstrate a performance advantage over the mean FA representation under the evaluated conditions. The bounded contribution is the separation of an implementation-dependent deficit from the absence of demonstrated incremental value after that sensitivity was addressed.

### 4.2. Implications for Healthcare Model Development and Validation

In the primary threshold-10 analysis, the design-only model had the most favorable mean point estimates across repeats, although paired conditional intervals for its comparisons with the interview-feature models included zero. This model used the adult-versus-pediatric cohort indicator, and the two strata differed in recruitment composition and screen-positive prevalence. In this cohort, the benchmark therefore illustrates how differences in outcome prevalence across study-design strata can contribute to discrimination without interview-derived features. This finding supports retaining explicit design-aware benchmarks under the same participant splits when multimodal interview models are evaluated. With the pediatric threshold changed to 11, absolute AP estimates were lower and the design-only benchmark no longer had the highest mean AP. Thus, its apparent advantage depended on the operational outcome definition, whereas the absence of demonstrated GMM incremental value persisted.

In the post hoc adult-only analysis, the adult-source FA model had more favorable AP, ROC-AUC, and Brier point estimates than the adult-source design-only model, although the restricted sample was small.

Discrimination metrics such as AP and ROC-AUC assess the ranking of screen-positive and screen-negative participants, whereas calibration assesses agreement between estimated probabilities and observed outcome frequencies. If estimated probabilities of same-session PHQ-9 screen-positive status were used to guide further assessment, underestimation could lead to missed opportunities for assessment, whereas overestimation could prompt unnecessary follow-up [29]. In this study, the low point estimates of the calibration slopes after cross-fitted recalibration (0.104 for FA and 0.093 for FA+GMM), together with their wide conditional confidence intervals, did not establish that these probabilities were sufficiently reliable for such decisions. Clinical evaluation should therefore assess the accuracy of probability estimates in the intended decision context alongside discrimination.

Considered together with the repeat-level incremental differences in Figure 1, these results indicate that increasing representation complexity alone is not a sufficient model-development strategy for AI-based depression assessment in healthcare. Complex representations should demonstrate incremental value over simpler, traceable representations and explicit design-aware benchmarks, with calibration assessed alongside discrimination.

The contribution of the primary finding is therefore an empirical model-selection boundary within the evaluated pipeline: the evaluated GMM summaries should not be prioritized over simpler mean representations solely because they encode additional distributional structure. This boundary provides a transparent basis for deciding which representations warrant further development while leaving open whether other FA–GMM or distributional specifications may add nonredundant interview-derived information. These findings provide model-selection and calibration evidence that can inform priorities for subsequent clinical evaluation.

The longer-term translational vision is a system capable of estimating depressive symptoms from a few minutes of naturally occurring conversation that is already taking place, such as in clinical care or counseling, and capable of providing supplementary screening information in healthcare and related support settings. The present structured protocol provides a controlled empirical foundation for evaluating this possibility.

Independent external validation should retain explicit design-aware benchmarks, assess calibration alongside discrimination, and provide sufficiently precise phase- and segment-retention-specific estimates. These next steps extend the present model-selection and validation contributions by addressing unresolved reproducibility, calibration, and interview-design questions.

### 4.3. Interview Phase and Segment-Retention Conditions

The prespecified phase-aware secondary analysis did not show improvement after GMM summaries were added. This analysis evaluated the four interview phases jointly and did not compare individual phases.

The separate phase-specific fixed-summary diagnostic yielded the highest point estimates for lifestyle but did not show consistent improvement after the GMM summaries were added. Because the phase summaries were precomputed before cross-validation and the analysis sets differed slightly across phases, these point estimates did not establish the superiority or practical utility of any individual phase.

The length-stability results varied nonmonotonically across phases and tested retained-segment conditions. The analysis did not identify a minimum retained-segment condition associated with stable performance. Taken together, these findings support prespecifying interview phase and segment-retention criteria as validation dimensions rather than treating them as interchangeable components of a whole-interview representation. These findings identify dimensions requiring explicit evaluation without establishing a clinically superior phase, an optimal segment-retention rule, or a corresponding speech duration.

Some length-stability cells contained few screen-positive participants, and the planned mixed-effects analyses were not estimable. Larger samples are therefore needed to estimate phase- and segment-retention-specific performance with sufficient precision and to determine whether a stable minimum retained-segment condition can be identified.

### 4.4. Methodological Interpretation

Exploratory fixed-summary diagnostics showed that the evaluated GMM summaries overlapped substantially with the participant-level mean FA scores: the four means explained 76.3% of the variance in the soft component-2 occupancy summary and 66.4% of the variance in the posterior assignment-margin summary. This descriptive redundancy offers a plausible explanation for why adding the posterior summaries did not improve performance, but it was not a fold-specific predictive comparison.

In the corresponding fixed-summary cross-validation sensitivity analysis, residualizing the GMM summaries within each training fold attenuated their performance deficit relative to the raw-summary model but did not improve point estimates for AP, ROC-AUC, or the Brier score beyond the fixed-summary FA model. This downstream-model diagnostic was consistent with substantial overlap between the GMM summaries and mean FA scores, but the precomputed representation summaries prevent this result from being interpreted as a full-pipeline internal-validation comparison.

Separately, the post hoc input-scaling sensitivity analysis showed improved performance after training-fold standardization relative to the native-scale FA+GMM model but did not produce clear incremental value beyond the FA model. The results therefore do not establish that segment-level distributional information is uninformative. Rather, they indicate that the two evaluated posterior summaries were not a useful addition under the present representation and validation pipeline.

FA–GMM remains a plausible low-complexity approach for representing segment-level heterogeneity within a traceable predictor structure. The present study shows how its contribution can be tested: posterior summaries should add nonredundant information beyond mean FA scores under identical participant splits, and their performance should remain interpretable after relevant preprocessing choices such as input scaling are examined.

### 4.5. Limitations

First, the sample was limited to 103 participants, including 24 with PHQ-9 scores of 10 or higher. Performance was evaluated using repeated nested cross-validation within the same cohort and was not externally validated. The conditional bootstrap intervals were based on the fixed split bank and generated out-of-sample predictions and did not propagate uncertainty from refitting the complete pipeline under alternative samples or split banks. The small number of positive outcomes limits the precision of model differences and calibration estimates; failure to demonstrate improvement does not exclude a benefit of a magnitude that this dataset could not resolve. The precision-based sample-size projections in Section S10 additionally condition on the empirical outcome/score distributions. They guide evaluation sample planning under those assumptions, but do not account for learning-curve effects or establish an adequate development sample size for the complete pipeline. The shared outer-CV recalibration diagnostic does not validate a deployable calibration procedure; its full training process would need evaluation within each outer-training set and subsequent independent validation.

Second, the outcome was same-session PHQ-9 screen-positive status rather than a future outcome. The primary analysis combined adult and pediatric strata, in which the J-PHQ-9 and threshold of 10 were used. Because criterion validity of the administered form and cutoff had not been established in pediatric participants, the pediatric classification is a study-specific operational label and should not be interpreted as a validated pediatric cutoff or clinical diagnosis. Refitting the models with a pediatric threshold of 11 evaluated sensitivity to that operational definition; it did not establish criterion validity for the administered form across ages 11–17. The adult-versus-pediatric cohort indicator also combined differences in age group, recruitment population, patient or student composition, and screen-positive prevalence. It should therefore be interpreted as a study-design variable rather than a pure age effect. Performance was not evaluated by sex, diagnosis, or treatment status. These limitations constrain interpretation of the pediatric labels and design-only model, as well as the transportability of absolute performance estimates. The FA-versus-FA+GMM contrast remains a matched comparison within the specified mixed cohort and operational outcome; it does not establish the same contrast for a clinically validated pediatric outcome or a differently composed cohort.

Third, the PHQ-9 was completed before the interview, and the fixed-structure videoconference interviews were conducted by qualified psychological counselors who were blinded to participants’ PHQ-9 scores and screen-positive status. The findings may not transport to routine clinical conversations, self-administered interviews, or other interviewer and protocol contexts. The approximately 30 min duration should not be interpreted as either a required or minimum duration for the envisioned application; shorter, naturally occurring conversations were not evaluated in this study. This study did not specify an intended user or operational probability threshold and did not evaluate a clinical workflow or effects on patient management; the reported metrics should be interpreted as model-development results.

Fourth, the primary conclusion is bounded by the evaluated representations. In the primary models, each feature block was compressed into one factor, participant-level aggregation relied mainly on weighted means, and the GMM extension comprised two posterior summaries. The native factor-score scales differed substantially, and the post hoc scaling analysis was not preregistered. Although standardization improved performance relative to the native-scale GMM specification, it did not establish incremental value beyond the FA model. The two-factor-per-modality and three-component-GMM sensitivity analyses examined only two specific alternative-complexity specifications and did not exhaust other distributional or temporal architectures. Feature-block, phase-specific fixed-summary, fixed-summary residualization, and retained-segment analyses were exploratory; some length-stability cells contained few screen-positive participants, and the planned mixed-effects analyses were not estimable. The fixed-summary analyses did not re-estimate FA or GMM within the cross-validation splits, and the length-stability analysis used 10 repeats and a restricted model set. These analyses do not establish the relative predictive contribution of individual feature blocks, an optimal phase, or an optimal segment-retention rule and corresponding speech duration.

Fifth, the text representation was based on transcripts generated with gpt-4o-transcribe and used without manual correction. No formal accuracy audit against the reference transcripts was conducted, and transcription error was not evaluated across participant subgroups. This limitation applies specifically to analyses involving text-derived features. The same qualitative finding regarding the incremental value of the evaluated GMM summaries was also observed in the prespecified analysis without the text representation (Section 3.4). This consistency does not quantify transcription error or rule out its influence on the four-representation results. Direct assessment against manually corrected reference transcripts, including subgroup-specific error estimates, would be valuable in future work to characterize and improve the linguistic representation.

Within these boundaries, the paired comparison using the same participants and split bank supports the within-pipeline model-selection conclusion that the evaluated GMM-derived summaries showed no clear incremental value beyond participant-level mean FA scores. The prespecified phase-aware analysis and the exploratory phase-specific fixed-summary and length-stability analyses separately support prespecifying interview phase and segment-retention criteria as validation dimensions; limited cell sizes constrain estimates of their optimal values, not the importance of evaluating them explicitly.

## 5. Conclusions

In this cohort comprising adult and pediatric participants, GMM posterior summaries did not demonstrate incremental value beyond participant-level mean FA scores. The preregistered native-scale FA+GMM model had less favorable mean performance estimates than FA in repeated internal validation. Post hoc training-fold standardization substantially attenuated this deficit, but the standardized GMM summaries showed no clear incremental value beyond participant-level mean FA scores. The two-factor-per-modality and three-component-GMM sensitivity specifications also did not show incremental benefit from the evaluated GMM summaries. The design-only model yielded the most favorable mean point estimates in the primary threshold-10 analysis, although its paired comparisons with the interview-feature models were imprecise; the interview-feature models also had low calibration slopes after cross-fitted recalibration. Together, these findings identify a model-selection boundary within the evaluated pipeline without excluding other distributional specifications: complex representations should demonstrate reproducible incremental value over simpler, traceable representations and explicit design-aware benchmarks, with calibration assessed alongside discrimination. The prespecified phase-aware analysis and the exploratory phase-specific fixed-summary and length-stability analyses further identify interview phase and segment-retention criteria as validation dimensions that should be prespecified, although they do not establish an optimal phase, retention rule, or input duration. These results provide an empirical basis for prioritizing representations and validation targets in subsequent multimodal depression-assessment research.

## Figures and Tables

**Table 1:** Participant characteristics by adult and pediatric strata.

Stratum	n	Age (Years), Median [Range]	Sex, n	PHQ-9, Median [IQR] (Range)	Screen-Positive, n (%)
Adult stratum	83	23.0 [19–53]	Male, 41; female, 38; missing, 4	3.0 [1.0–7.0] (0–18)	15 (18.1)
Pediatric stratum	20	14.5 [11–17]	Male, 11; female, 9	9.0 [5.0–11.25] (2–16)	9 (45.0)

IQR, interquartile range; PHQ-9, Patient Health Questionnaire-9. Age and sex were missing for four participants in the adult stratum; the corresponding age and sex summaries are therefore based on available values. Screen-positive status was defined using the prespecified PHQ-9 threshold of 10 in both strata. The adult stratum comprised university students and adult patients; their detailed breakdown is provided in Table S2.

**Table 2:** Participant-level estimation performance of primary analysis models and design-only model.

Model	Repeats	AP	ROC-AUC	Brier Score
*A. Mean performance across evaluable repeats*				
Design-only model (cohort indicator only)	25	0.332	0.612	0.169
Primary four-representation FA model	25	0.302	0.576	0.177
Primary four-representation FA+GMM model	24	0.282	0.546	0.179
*B. Participant-mean probabilities: Estimate [95% conditional interval]*				
Design-only model	25	0.356 [0.264, 0.547]	0.611 [0.464, 0.752]	0.169 [0.156, 0.182]
FA model	25	0.327 [0.255, 0.501]	0.647 [0.527, 0.770]	0.176 [0.173, 0.179]
FA+GMM model	24	0.279 [0.229, 0.397]	0.603 [0.479, 0.729]	0.178 [0.175, 0.180]

Note. AP, average precision; Brier, Brier score; FA, factor analysis; GMM, Gaussian mixture model; ROC-AUC, area under the receiver operating characteristic curve. Evaluable repeats required complete participant-level predictions and are not independent external validations. One FA+GMM outer fit failed because an inner-fold logistic model did not converge after the specified retry; this event was recorded as a method failure, and the affected repeat was excluded from the model summary. Both interview-feature models include the prespecified adult-versus-pediatric stratum indicator. The no-skill AP baseline is 24/103 = 0.233. Higher AP and ROC-AUC and lower Brier scores are better. Panel B evaluates one mean out-of-sample probability per participant and uses 2000 outcome-stratified participant bootstrap resamples. Its estimates and intervals describe that aggregation, not the repeat means in Panel A; model rankings can also differ between the two aggregations. The intervals condition on the generated predictions and do not include full-pipeline refitting.

**Table 3:** Paired differences in the primary comparison and design-only model comparison.

Comparison	Paired Repeats	ΔAP	ΔROC-AUC	ΔBrier
*A. Mean of paired differences across repeats*				
FA+GMM − FA	24	−0.020 [−0.046, +0.001]	−0.031 [−0.053, −0.008]	+0.0016 [0.00043, 0.00294]
FA − Design-only	25	−0.031	−0.036	+0.0085
FA+GMM − Design-only	24	−0.051	−0.066	+0.0101
*B. Within-participant means across repeats: Paired conditional estimate [95% interval]*				
FA+GMM − FA	24	−0.048 [−0.147, −0.008]	−0.046 [−0.083, −0.011]	+0.00153 [+0.00041, +0.00281]
FA − Design-only	25	−0.029 [−0.198, +0.142]	+0.036 [−0.128, +0.221]	+0.00762 [−0.00504, +0.02032]
FA+GMM − Design-only	24	−0.079 [−0.248, +0.058]	−0.011 [−0.173, +0.171]	+0.00911 [−0.00352, +0.02188]

Note. AP, average precision; Brier, Brier score; FA, factor analysis; GMM, Gaussian mixture model; ROC-AUC, area under the receiver operating characteristic curve. Positive differences in AP and ROC-AUC favor the model on the left; negative differences in Brier score favor the model on the left. Panel A summarizes the mean of the paired repeat-level differences. For its primary FA+GMM-minus-FA row, brackets give the post hoc conditional 95% interval from 2000 ordinary participant bootstrap samples, retaining each participant’s complete prediction vector and recomputing paired metrics within repeats before averaging (Section S10). In Panel A, conditional 95% intervals were calculated only for the primary FA+GMM-minus-FA comparison; they were not calculated for comparisons with the design-only model. Panel B first averages each participant’s out-of-sample estimated probability across the repeats evaluable for both models and then reports the paired metric difference and its outcome-stratified participant-level bootstrap interval. The panels therefore use different summary bases. Intervals in both panels are conditional on the prespecified split bank and already-generated out-of-sample predictions and do not represent full-pipeline or external-validation uncertainty.

**Table 4:** Participant-level metrics before and after cross-fitted recalibration.

Model	Repeats	AP	ROC-AUC	Brier	Intercept Mean (SD) [95% CI]	Slope Mean (SD) [95% CI]
*A. Raw out-of-sample probabilities*						
Design-only	25	0.332	0.612	0.169	0.474 (0.000) [−1.007,2.020]	1.412 (0.000) [0.248,2.746]
FA	25	0.302	0.576	0.177	0.354 (2.289) [−2.808,3.498]	1.304 (1.924) [−1.256,3.884]
FA+GMM	24	0.282	0.546	0.179	−0.745 (2.577) [−3.932,2.264]	0.372 (2.160) [−2.181,2.853]
*B. Cross-fitted recalibrated probabilities*						
Design-only	25	0.323	0.611	0.168	−0.036 (0.000) [−1.151,1.111]	0.970 (0.000) [0.158,1.896]
FA	25	0.279	0.540	0.182	−1.068 (1.122) [−2.692,0.563]	0.104 (0.941) [−1.138,1.477]
FA+GMM	24	0.263	0.515	0.185	−1.082 (0.815) [−2.757,0.661]	0.093 (0.680) [−1.238,1.581]

Note. AP, average precision; Brier, Brier score; CI, confidence interval; FA, factor analysis; GMM, Gaussian mixture model; ROC-AUC, area under the receiver operating characteristic curve. AP, ROC-AUC, and Brier are means across evaluable repeats; each repeat contains 103 participants, including 24 screen-positive participants. Calibration coefficients assess pooled validation-fold predictions within each repeat and are summarized as mean (SD) across repeats. The SD describes split-related variation, not a standard error or confidence interval. Brackets give conditional 95% percentile confidence intervals for the mean coefficients from 2000 participant-level bootstrap samples, retaining all predictions from each sampled participant together across repeats. Predictions and recalibration transformations were fixed. The intercept was estimated jointly with the slope. Panel B reports evaluation coefficients for the cross-fitted recalibrated probabilities, not the coefficients used to transform individual folds. Because recalibration was performed using leave-one-outer-fold-out cross-fitting, different recalibration coefficients were applied to each validation fold. Therefore, recalibrated AP and ROC-AUC values are reference values based on pooled estimated probabilities after fold-specific recalibration rather than ranking metrics that would result from applying the same monotone transformation to all targets. Higher AP and ROC-AUC values and lower Brier scores are better.

**Table 5:** Mean estimation performance across repeats in the exploratory fixed-summary comparison by interview phase.

Phase	*n*	FA Raw AP	FA Raw ROC-AUC	FA+GMM Raw AP	FA+GMM Raw ROC-AUC	ΔAP	ΔROC-AUC
happy	103	0.287	0.539	0.272	0.514	−0.015	−0.025
sad	103	0.271	0.525	0.276	0.513	+0.005	−0.012
anger	102	0.261	0.513	0.248	0.485	−0.014	−0.027
lifestyle	102	0.335	0.652	0.326	0.628	−0.009	−0.024

Note. AP, average precision; FA, factor analysis; GMM, Gaussian mixture model; ROC-AUC, area under the receiver operating characteristic curve. Values are means across 25 evaluable repeats. Δ denotes three-representation FA+GMM minus three-representation FA. Precomputed phase-specific, duration-weighted participant summaries from run initial_qc_v6 were supplied to nested cross-validation of the downstream ridge logistic model; FA and GMM were not re-estimated within the inner or outer splits. These values are therefore exploratory fixed-summary diagnostics and not full-pipeline phase-specific internal-validation estimates. Each phase included 24 screen-positive participants; the no-skill AP baselines were 24/103 = 0.233 for happy and sad and 24/102 = 0.235 for anger and lifestyle. Higher AP and ROC-AUC values are better.

## Data Availability

The raw audio, video, and verbatim transcript data are not publicly available because the interview content may permit participant identification and the dataset includes minor participants. Access requests will be considered individually by the corresponding author, subject to institutional approval, ethical restrictions, and appropriate data-use conditions. An anonymous-review version of the analysis code is available through the following view-only OSF link: https://osf.io/xb4te/overview?view_only=061da9f3e5374cb796c0cbb5f30eaa6f (accessed on 11 October 2026).

## Supplementary Materials

The following supporting information can be downloaded at: https://www.mdpi.com/article/10.3390/healthcare14213602/s1, File S1, supplementary section with implementation details, model diagnostics, secondary and sensitivity analyses, reproducibility information, and the post hoc precision and pediatric outcome-threshold analyses.

## Abbreviations

The following abbreviations are used in this manuscript:

AI	artificial intelligence
AP	average precision
FA	factor analysis
GMM	Gaussian mixture model
IQR	interquartile range
J-PHQ-9	Japanese version of the Patient Health Questionnaire-9
MINRES	minimum residual
PHQ-9	Patient Health Questionnaire-9
PHQ-A	Patient Health Questionnaire for Adolescents
ROC-AUC	area under the receiver operating characteristic curve
