# Supplementary material for: Multimodal AI for Same-Session Estimation of PHQ-9 Screen-Positive Status from Counselor-Conducted Fixed-Structure Interviews: A Preregistered Internal Validation Study of Incremental Value and Calibration
Source: Healthcare (Basel). 2026 Oct 23;14(21):3602. doi: 10.3390/healthcare14213602 (PMC13762850; doi:10.3390/healthcare14213602)
Supplement: Supplementary file 1 [file healthcare-14-03602-s001.zip › healthcare-4615206-supplementary.pdf]

Supplementary Materials: Multimodal AI for Same-Session Estimation of PHQ-9 Screen-Positive Status from Counselor-Conducted Fixed-Structure Interviews: A Preregistered Internal Validation Study of Incremental Value and Calibration

Keita Kiuchi, Kotaro Kashihara, Toshiki Takanabe, Hidehiro Umehara, Koushi Irizawa, Masahito Nakataki, Shusuke Numata, Xin Kang, Minoru Yoshida and Kazuyuki Matsumoto

This supplementary material provides technical details supporting the evaluation of a multimodal artificial intelligence pipeline for same-session estimation of PHQ-9 screen-positive status. The source interviews were conducted by qualified psychological counselors according to a fixed structure. The administered instrument was the Japanese version of the Patient Health Questionnaire-9 (J-PHQ-9), validated by Muramatsu et al. [1].

S1. Details of the Primary Analysis Pipeline and Validation Design

Analysis Scope and Fixed Design

This supplementary material provides additional implementation details for the Methods section of the main text. The unit of inference in the primary analysis was the participant, and speech segments served as internal units for feature extraction and participant-level aggregation. The analytic sets, number of factors, and number of Gaussian mixture model (GMM) components were fixed in advance based on data availability, quality control (QC), and model complexity rather than optimized for predictive performance.

Transcript Source and Representation Alignment

Transcripts of participant speech generated with gpt-4o-transcribe were used throughout the analysis. In the primary analysis, the openSMILE, HuBERT, PyFeat, and text representations were aligned to the same speech segments by exact matching on normalized participant ID and start and end times. Topic anchors specified in seconds were read as strings, converted to `Decimal` values, multiplied by 1000, and rounded half up to the nearest millisecond. Overlap-based and tolerance-based joins for frame-level features were limited to sensitivity analyses and were not used in the primary analysis.

Segment-Level Video and Text Processing

For PyFeat, segment-level summaries were created from frame-level outputs. Frames with PyFeat face-detection confidence of 0.90 or higher were treated as valid frames, and segments with fewer than 5 valid frames were treated as missing. The aggregation targets comprised head pose (pitch, roll, yaw), selected action units, and selected expression probabilities (happiness, sadness, and neutral). The mean and standard deviation of each metric were calculated, yielding one PyFeat vector per segment. Text representations were created only from participant speech. Processing included normalization using Unicode Normalization Form Compatibility Composition (NFKC), trimming leading and trailing whitespace, collapsing consecutive whitespace, and standardizing obvious nonverbal tags. Sentence embeddings were treated as missing for segments that were empty, contained only whitespace, or contained only nonverbal tags.

Standardization, Subsampling, and Factor Analysis

Within each inner- and outer-training split, columns were standardized separately by feature representation, and zero-variance columns were removed. To prevent participants with many speech segments from disproportionately influencing factor analysis (FA) and GMM fitting, participant-balanced subsampling was performed with a maximum of 200 segments per participant. FA was performed separately for the openSMILE, HuBERT, PyFeat, and text feature representations, with the number of factors fixed at one for each. Factor loadings were estimated using unrotated minimum residual (MINRES) extraction, and factor scores for each segment were estimated using regression scoring [2]. Inner-validation and outer-test participants were transformed using the parameters estimated in their corresponding training split. The inner fits were used for penalty selection; the outer-training fit supplied the final held-out predictions. The number of factors was fixed rather than selected using the validation or test outcomes.

Sign Alignment of Factor Scores

Because the signs of factor scores and loadings in one-factor FA can flip across folds, label-independent sign alignment was performed for each training split. For openSMILE, HuBERT, and PyFeat, the raw feature with the largest absolute loading was used as the anchor; when the anchor loading was negative, the signs of the factor scores and the loading vector were flipped together. For the text factor, a lexical negativity index was calculated as the sum of the z-scores for the negation-expression rate, absolutist-expression rate, negative-emotion-word rate, and symptom-cue-word rate minus the z-score for the positive-emotion-word rate. If the correlation between this index and the text factor score was negative, the factor-score sign was flipped.

GMM Fitting and Participant-Level Summaries

The GMM was fitted in the four-dimensional space formed by concatenating the four sign-adjusted segment-level factor scores from openSMILE, HuBERT, PyFeat, and text. In the primary analysis, the settings were fixed at two components, diagonal covariance matrices, k-means initialization, 20 initializations, covariance regularization of 10^{-5}, and a maximum of 500 iterations. A GMM solution was accepted if it converged, each component contained at least 5% of training segments, and each component included segments from at least 5 training participants, with segment assignments determined by maximum posterior probability. If these criteria were not met or convergence failed with covariance regularization of 10^{-5}, the regularization value was deterministically increased to 10^{-4} and then 10^{-3}.

The four factor-score dimensions were entered into each GMM on their fold-specific native scales, without additional cross-representation z-standardization. This scale handling was part of the prespecified primary implementation. Participant-level predictors were standardized within each training split before fitting the ridge logistic regression.

The GMM-derived participant-level features were the segment-length-weighted mean posterior probability of membership in ordered component 2 (a soft component-2 occupancy summary) and the similarly weighted mean difference between the largest and second-largest posterior probabilities (a posterior assignment-margin summary). Component order was determined within each training fold by treating the component with the smaller sum of sign-aligned factor-score means across the four feature representations as component 1 and the component with the larger sum as component 2; lexicographic ordering was used only to break exact ties. Neither summary is a conventional measure of distributional skewness, and ordered component 2 should not be interpreted as a clinical subtype.

For clarity, the runtime identifiers `fa4_text` and `fagmm4_text` denote the four-representation branch, in which text is one of the four representations; the suffix `_text` does not indicate an additional text predictor. The corresponding reporting labels are the FA model and FA+GMM model, respectively; both include the cohort indicator. The adult-only sensitivity analysis used analogous runtime identifiers: `adult_fa4_text` for FA and `adult_fagmm4_text` for FA+GMM.

Cross-Validation and Performance Metrics

Stratification used the cross-classification of phq9_binary × adult_child as the default rule and was simplified to phq9_binary alone only when the number of participants in any stratum was less than max(n_splits, 5). Because each validation fold in the 5-fold outer cross-validation (CV) contained only approximately 4–5 screen-positive participants, performance comparisons from a single split could be unstable. Accordingly, this study used mean performance across repeats and paired differences from repeated nested cross-validation under identical splitting conditions as the main reported quantities, rather than results from individual splits or dichotomous decisions based on p values. Averaging over 25 repeats reduced the influence of split-induced variability; it increased neither the number of independent participants nor the number of independent external validation sets.

All three primary-analysis models used logistic regression with an unpenalized intercept, an L_2 penalty on the coefficients, no class weights, and the lbfgs solver (maximum of 10,000 iterations; tolerance 10^{-8}). The penalty parameter λ was selected within each outer-training fold from 25 logarithmically spaced values between 10^{-4} and 10^{2}. Selection minimized mean inner-fold log loss with the one-standard-error rule; among values within one standard error of the minimum, the largest λ was selected. The implemented objective was mean log loss plus $\lambda\|\boldsymbol{\beta}\|_2^2/2$, with the intercept unpenalized.

Average precision (AP) and the area under the receiver operating characteristic curve (ROC-AUC) were calculated using the raw out-of-sample estimated probabilities for participants in the outer validation folds, and stepwise average precision was used for AP. The trapezoidal area under the precision-recall curve (PR-AUC) was not used as the primary metric. Recalibrated AP and ROC-AUC are also shown as reference values. Because leave-one-outer-fold-out cross-fitting estimated different intercepts and slopes for the validation folds, these values should not be interpreted as pure ranking metrics after monotone calibration. They are auxiliary descriptive metrics that incorporate fold-specific recalibration.

Recalibration reused the five outer-fold assignments without a new random partition or additional stratification. For each held-out fold, a logistic intercept and slope were fitted to the raw out-of-fold probabilities and labels from the other four folds. Separate evaluation coefficients were then fitted to the pooled cross-fitted probabilities within each repeat. The reported intercept was estimated jointly with the slope, rather than with a fixed unit-slope offset. The models generating the other folds' probabilities had overlapping training sets, including participants in the fold being recalibrated. Consequently, this post-processing diagnostic is not a fully nested validation of a recalibrated prediction pipeline. Raw model performance and recalibrated diagnostics are reported separately in the main text.

Conditional Confidence Intervals for Calibration Coefficients

In a post hoc analysis, 95% percentile confidence intervals were calculated for the mean of the repeat-specific calibration intercepts and slopes in main-text Table 4. We drew 2,000 ordinary bootstrap samples of 103 participants with replacement (random seed 20260919). Each draw retained a participant's complete set of predictions across models, probability types, and repeats. Outcome counts were allowed to vary; this differs from the outcome-stratified bootstrap used for AP, ROC-AUC, and Brier score. The original evaluable repeat sets were retained: 25 for design-only and FA and 24 for FA+GMM, excluding Repeat 8 for the latter.

For each bootstrap sample and repeat, we refitted the joint intercept and slope assessment regression using the sampled participants, then averaged the coefficients across repeats. The 2.5th and 97.5th percentiles of these bootstrap means supplied the intervals. The regression retained the original calibration routine's weak slope penalty ($C = 10^6$) and unpenalized intercept; probabilities were clipped to $[10^{-12}, 1 - 10^{-12}]$ before the logit transformation. A batched Newton implementation of the same objective was checked against the original scikit-learn fits. All 2,000 samples completed without fit exclusions or alternative estimators.

Raw and reconstructed recalibrated predictions were held fixed. These conditional descriptive intervals account for resampling participants while retaining their repeated predictions together. They do not include uncertainty from fitting the prediction models or recalibrators, changing the split bank, or dependence between participants induced by overlapping training sets. They therefore do not provide full-pipeline uncertainty intervals.

Secondary, Sensitivity, and Exploratory Analyses

In the prespecified phase-aware secondary analysis, the 102 participants in `PA*` with analyzable data for all four phases were used to compare the phase-aware design-only benchmark, phase-aware FA, and phase-aware FA+GMM models on the same split bank. In the exploratory length-stability sensitivity analysis, we deterministically formed participant sets for each interview phase and retained-segment condition ($N = 16, 32, 48$), retaining the first N complete four-representation segments in chronological order for each participant. We compared an FA+GMM prefix model, for which participant summaries were constructed from only the first N segments, with a same-phase full FA+GMM model, which used all segments from the same phase in the same participant set. This analysis estimated only the prefix-minus-full gap to limit the computational burden. The corresponding FA prefix model was not evaluated.

S2. Primary-Analysis Diagnostics and Analytic-Set Descriptions

S2a. One-Factor FA and Lexical Correspondence Diagnostics

Table S1. Cross-Validation Summary of One-Factor FA Diagnostics and the Lexical Sidecar Dictionary

The upper panel summarizes one-factor FA diagnostics from 125 outer fits of `fa4_text_secondary`. Loadings are reported as absolute values after sign alignment, with each loading summary shown as the median across outer fits (minimum–maximum). The factor-score standard deviation (SD), calculated across all evaluable segments in each outer fit, serves as a diagnostic of score-scale stability across folds. Because regression-based factor-score scales are not directly comparable across representations, the reported SDs also document the numerical scales that entered the primary GMM; no cross-representation z-standardization was applied before GMM fitting.

Panel A. One-Factor FA Diagnostics Across Outer Fits

Feature representation	Original dimensions / retained dimensions	Outer fits	Training segments / evaluation segments	Sign-alignment anchor	Sign flips	Maximum absolute loading	Median absolute loading	Factor-score SD
openSMILE	62 / 62	125	11,393–12,660 / 17,522	`loudness_sma3_amean` in 124/125	0/125	0.8588 (0.8431–0.8783)	0.2876 (0.2347–0.3161)	35.658 (23.451–51.272)
HuBERT	768 / 768	125	11,393–12,660 / 17,522	`hubert_0148` in 68/125, `hubert_0724` in 57/125	81/125	0.7814 (0.7574–0.8262)	0.2526 (0.2405–0.2664)	0.975 (0.938–1.014)
PyFeat	30 / 30	125	11,300–12,546 / 17,164	`au06_sd` in 63/125, `au06_mean` in 34/125, `au12_mean` in 28/125	83/125	0.8149 (0.7933–0.8445)	0.2602 (0.2167–0.2918)	0.944 (0.908–0.994)
Text	768 / 768	125	11,389–12,655 / 17,504	Correlation with lexical negativity index	125/125	0.7915 (0.7833–0.8034)	0.2761 (0.2707–0.2818)	1.030 (1.014–1.044)

Note. The one-factor FA loading summary is a descriptive diagnostic used to assess whether a central common axis was estimated for each feature representation; it represents neither the proportion of variance explained in principal component analysis (PCA) nor the retention of depression-related information. For openSMILE, HuBERT, and PyFeat, signs were aligned using the feature with the largest absolute loading as the anchor. For text, the sign was aligned to ensure a positive correlation with the lexical negativity index. This index was calculated as the sum of the z-scores for the negation-expression, absolutist-expression, negative-emotion-word, and symptom-cue-word rates minus the z-score for the positive-emotion-word rate. FA, factor analysis; GMM, Gaussian mixture model; PCA, principal component analysis; SD, standard deviation.

The lower panel lists the contents of the lexical sidecar dictionary used for sign alignment and interpretive support of the text factor. The text factor was estimated by applying one-factor FA to 768-dimensional sentence embeddings obtained from participant speech. The dictionary was constructed from Japanese source terms, but the table reports those terms in Hepburn-style romanization to avoid mixing Japanese script into the supplementary material. Some entries are substring stems used for dictionary matching rather than standalone words; therefore, the romanized strings should be read as source-language matching cues, not as formal English translations. Repeated romanized forms correspond to multiple original spellings in the source dictionary.

Panel B. Lexical Sidecar Dictionary Used for Text-Factor Sign Alignment and Interpretive Support

Category	Item	Romanized source terms	Interpretation and notes
Lexical correspondence	Negation expressions	`janai`, `dewa-nai`, `nai`, `nakatt-`, `naku`, `nu`, `masen`, `nai`, `dekinai`	Approximate gloss: not, no, absent, or cannot. Included positively in the lexical negativity index
Lexical correspondence	Absolutist expressions	`itsumo`, `nanimo`, `zenzen`, `zenbu`, `kanzen`, `kanarazu`, `zettai`	Approximate gloss: always, nothing, at all, all, complete, certainly, or absolute. Included positively in the lexical negativity index
Lexical correspondence	Negative-emotion and anxiety words	`shindoi`, `tsurai`, `fuan`, `iya`, `sabishii`, `shinpai`, `ikari`, `okoru`, `kowai`, `kanashii`, `muryokukan`, `kincho`, `kurushii`, `ochikomu`, `tsurai`	Approximate gloss: distress, pain, anxiety, dislike, loneliness, worry, anger, fear, sadness, helplessness, tension, suffering, or feeling down. Included positively in the lexical negativity index
Lexical correspondence	Positive emotion words	`ureshii`, `yokatta`, `yorokobu`, `suki`, `ureshii`, `anshin`, `shiawase`, `tanoshii`, `omoshiroi`	Approximate gloss: glad, good, joyful, liking, relief, happiness, fun, or interesting. Subtracted when calculating the lexical negativity index
Lexical correspondence	Symptom-cue words	`shindoi`, `darui`, `kachi`, `hakike`, `tanoshikunai`, `kiryoku`, `tsukare`, `tsukareru`, `nemui`, `nemure`, `suimin`, `kyomi`, `shuchu`, `zutsu`, `tabere`, `shokuyoku`	Approximate gloss: fatigue, sluggishness, worth, nausea, not enjoyable, energy, tiredness, sleepiness, sleep, interest, concentration, headache, eating, or appetite. Included positively in the lexical negativity index
Lexical correspondence	First-person singular words	`atashi`, `ore`, `boku`, `washi`, `watashi`, `ore`, `boku`, `watashi`, `jibun`	Approximate gloss: first-person singular self-references such as I, me, or myself. Retained as an auxiliary person category; not included in the main sign-alignment index

Note. `intfloat/multilingual-e5-base` is a publicly available multilingual sentence-embedding model. In this study, the model was used without updating its weights to convert participant speech into 768-dimensional sentence embeddings.

S2b. Cohort Composition and PHQ-9 Distribution

Table S2. Distribution of PHQ-9 Screen-Positive Status by Two-Group Cohort Indicator and Operational Three-Group Breakdown

This table shows the distribution of PHQ-9 `screen-positive` and `screen-negative` labels in the common set for the primary analysis ($n = 103$) according to both the two-group cohort indicator used as a fixed covariate and the operational three-group breakdown. The two-group cohort indicator should be interpreted as a coarse design variable that groups university students and adult patients into the adult stratum and pediatric patients into the pediatric stratum.

Operational group	Two-group cohort indicator	Screen-negative	Screen-positive	Total	Screen-positive rate
University students	Adult stratum	28	6	34	17.6%
Adult patients	Adult stratum	40	9	49	18.4%
Adult stratum subtotal	Adult stratum	68	15	83	18.1%
Pediatric patients	Pediatric stratum	11	9	20	45.0%
Total	-	79	24	103	23.3%

Note. J-PHQ-9 denotes the Japanese version of the Patient Health Questionnaire-9 (PHQ-9).

The screen-positive proportions were 15/83 (18.1%) in the adult stratum and 9/20 (45.0%) in the pediatric stratum. In a post hoc outcome-threshold sensitivity analysis, retaining the threshold of 10 in the adult stratum and applying the threshold of 11 reported for adolescents in the pediatric stratum reclassified two pediatric participants with PHQ-9 scores of 10 as screen-negative. The screen-positive count in the pediatric stratum consequently decreased to 7/20 (35.0%), and the overall count decreased to 22/103 (21.4%). Six models were refitted under this alternative definition using the original nested-cross-validation partitions (Section S11). The threshold of 11 was treated as an alternative reference, not as a validated cutoff for the full pediatric age range of 11–17 years.

S2c. Age, Sex, and PHQ-9 Distribution by Cohort

Table S3. Age, Sex, PHQ-9 Distribution, and Screen-Positive Status in the Adult and Pediatric Strata

Age and sex were obtained from the questionnaire-derived feature table used as an input to the analysis. Because age and sex were missing for four participants in the adult stratum, age summaries and sex distributions are based on available values. There were no missing age or sex values in the pediatric stratum.

Two-group cohort indicator	n	Age, median [range]	Sex	PHQ-9, median [IQR] (range)	Screen-positive, n (%)
Adult stratum	83	23.0 [19–53]	Male, 41; female, 38; missing, 4	3.0 [1.0–7.0] (0–18)	15 (18.1)
Pediatric stratum	20	14.5 [11–17]	Male, 11; female, 9	9.0 [5.0–11.25] (2–16)	9 (45.0)

Note. IQR, interquartile range; J-PHQ-9, Japanese version of the Patient Health Questionnaire-9; PHQ-9, Patient Health Questionnaire-9.

Pediatric outcome-measure provenance. The pediatric participants completed the same J-PHQ-9 as the adults; the Patient Health Questionnaire for Adolescents (PHQ-A) was not administered. Participants generally completed the questionnaire themselves. Researchers or study staff clarified items and assisted as needed, but participants, rather than proxy respondents, provided the scored responses. The common threshold of 10 defined the prespecified outcome; pediatric-specific Japanese criterion validity for this threshold has not been established. Accordingly, pediatric screen-positive status was treated as a study-specific operational label, and the design-only adult–pediatric comparison should be interpreted accordingly. The post hoc threshold-11 analysis reclassified two participants, and the resulting model-performance estimates are reported in Section S11. This sensitivity analysis did not establish pediatric criterion validity.

S2d. Mixed-Effects Estimation Status

Table S4. Estimation Status of Mixed-Effects Models by Endpoint

Endpoint	n_rows	n_subjects	Status	Main reason
Soft component-2 occupancy summary	410	103	Not estimable	Non-positive-definite Hessian; random-effect variance singular
Text factor	410	103	Not estimable	Non-positive-definite Hessian; random-effect variance singular
Posterior assignment-margin summary	410	103	Not estimable	Non-positive-definite Hessian; random-effect variance singular
openSMILE factor	410	103	Not estimable	Non-positive-definite Hessian; random-effect variance singular
HuBERT factor	410	103	Not estimable	Non-positive-definite Hessian; random-effect variance singular
PyFeat factor	410	103	Not estimable	Non-positive-definite Hessian; random-effect variance singular
Additional candidate metrics	0	0	Not evaluated	Insufficient input columns

Note. `n_rows` and `n_subjects` denote the numbers of rows and unique participants available for the attempted endpoint model, respectively. "Not estimable" indicates that model fitting was attempted but did not yield an admissible estimate; "Not evaluated" indicates that the required input columns were unavailable.

S3. Secondary and Auxiliary Analyses

This section presents the prespecified secondary analyses and the sensitivity and exploratory analyses used to examine boundary conditions of the primary result.

S3a. `S*` Supporting, Primary, and Alternative-Complexity Analyses

Table S5. Performance of the Supporting, Primary, and Alternative-Complexity `S*` Model Families

Analysis family	Model	Repeats	Raw AP	Raw ROC-AUC	Raw Brier score	Recal. AP	Recal. ROC-AUC	Recal. Brier score
Supporting three-representation	Supporting `S*` FA (`fa3`)	25	0.336	0.618	0.175	0.319	0.601	0.182
Supporting three-representation	Supporting `S*` FA+GMM (`fagmm3`)	25	0.299	0.581	0.178	0.275	0.539	0.185
Primary four-representation	Primary four-representation FA, current text (`fa4_text`)	25	0.302	0.576	0.177	0.279	0.540	0.182
Primary four-representation	Primary four-representation FA+GMM, current text (`fagmm4_text`)	24	0.282	0.546	0.179	0.263	0.515	0.185
Two-factor-per-modality sensitivity (`cap2`)	Four-representation FA (`fa4_text_cap2`)	25	0.303	0.558	0.177	0.281	0.535	0.182
Two-factor-per-modality sensitivity (`cap2`)	Four-representation FA+GMM (`fagmm4_text_cap2`)	25	0.287	0.549	0.178	0.270	0.515	0.184
Three-component-GMM sensitivity (`K=3`)	Primary four-representation FA comparator (reused; `fa4_text`)	25	0.302	0.576	0.177	0.279	0.540	0.182
Three-component-GMM sensitivity (`K=3`)	Four-representation FA+GMM (`fagmm4_text_k3`)	25	0.268	0.521	0.179	0.253	0.497	0.184

Note. `S*` is the 103-participant analysis set used for the supporting, primary, and alternative-complexity analyses. AP, average precision; CV, cross-validation; FA, factor analysis; GMM, Gaussian mixture model; Recal., recalibrated; ROC-AUC, area under the receiver operating characteristic curve. All rows used the primary participant split bank, fold-specific representation fitting, and the complete-repeat rule. The supporting and primary rows were generated by the Step09 nested-CV pipeline; the `cap2` and `K=3` rows were generated by the corresponding Step12 nested-CV sensitivity pipeline. In `cap2`, each modality had a fixed two-factor capacity, with the second factor set to zero when only one factor was estimable. The `K=3` FA+GMM model summarized the ordered second- and third-component posterior probabilities and the posterior assignment margin. Because this sensitivity changed only the number of GMM components, its FA comparator reused the primary `fa4_text` model. The primary four-representation rows are repeated only to document their provenance and therefore match Table 2 of the main text exactly for raw metrics and Table 4 of the main text exactly for recalibrated metrics. One primary FA+GMM repeat was excluded as a recorded method failure, as described in the main text. Higher AP and ROC-AUC values and lower Brier scores are better. Detailed provenance is given in Table S14.

Across both alternative-complexity sensitivities, the FA comparator had more favorable mean point estimates than the corresponding FA+GMM model for raw and recalibrated AP, ROC-AUC, and Brier score. For `cap2`, the mean FA+GMM-minus-FA differences were ΔAP -0.015, ΔROC-AUC -0.009, and ΔBrier $+0.0006$ for raw metrics. For `K=3`, the corresponding differences were ΔAP -0.033, ΔROC-AUC -0.054, and ΔBrier $+0.0018$.

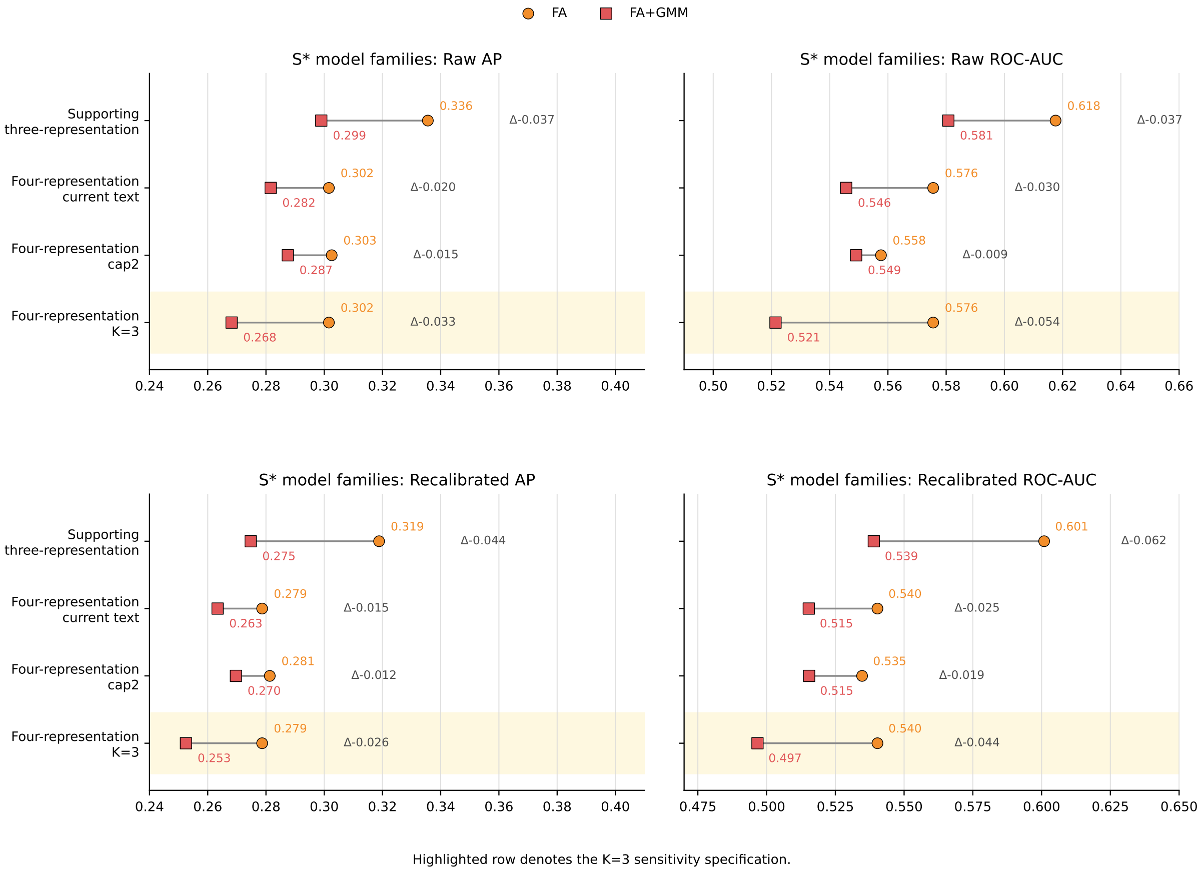

Figure S1. Raw and recalibrated average precision (AP) and area under the receiver operating characteristic curve (ROC-AUC) for the factor analysis (FA) and FA plus Gaussian mixture model (FA+GMM) specifications in the supporting, primary, and alternative-complexity `S*` model families. The `K=3` comparison reuses the primary four-representation FA model because only GMM complexity was changed.

S3b. Phase-Aware Prespecified Secondary Analysis

Table S6. Participant-Level Metrics for the Phase-Aware Prespecified Secondary Analysis in `PA*`

Model	Repeats	Raw AP	Raw ROC-AUC	Raw Brier score	Recal. AP	Recal. ROC-AUC	Recal. Brier score
Phase-aware design-only benchmark	25	0.339	0.624	0.170	0.339	0.624	0.170
Phase-aware FA	25	0.269	0.533	0.180	0.252	0.488	0.183
Phase-aware FA+GMM	25	0.261	0.519	0.180	0.243	0.476	0.185

Note. All models were evaluated in `PA*`, which included the 102 participants with analyzable data for all four phases. The phase-aware design-only benchmark used only the cohort indicator; phase-aware FA and phase-aware FA+GMM denote the corresponding feature models. Recal. denotes recalibrated. AP, average precision; FA, factor analysis; GMM, Gaussian mixture model; ROC-AUC, area under the receiver operating characteristic curve. Higher AP and ROC-AUC values and lower Brier scores are better.

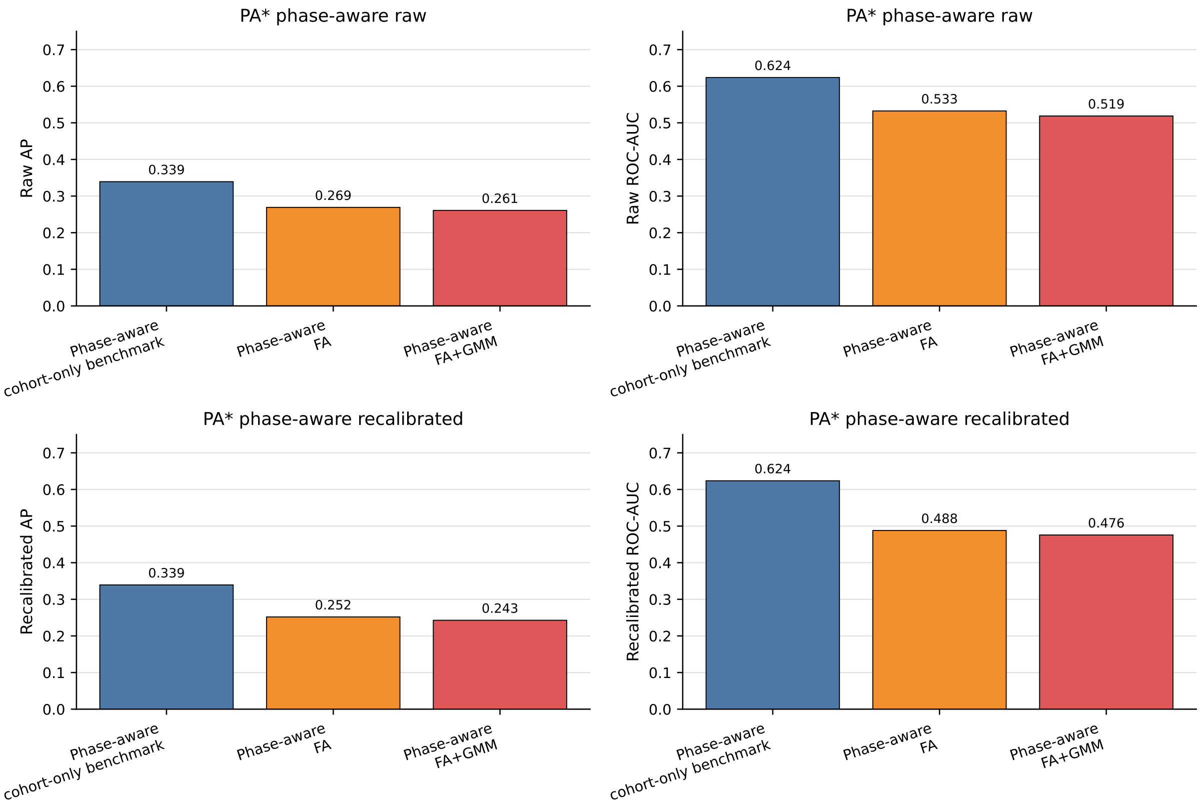

Figure S2. Raw and recalibrated average precision (AP) and area under the receiver operating characteristic curve (ROC-AUC) for the design-only, factor analysis (FA), and FA plus Gaussian mixture model (FA+GMM) models in `PA*`.

S3c. Text Representation Sensitivity

Table S7. Participant-Level Metrics for Text Representation Sensitivity in `T*`

Model	Target n	Mean evaluable participants per repeat	Evaluable repeats	Raw AP	Raw ROC-AUC	Raw Brier score
`T*` text-sensitivity FA model (current text)	103	96.4	25	0.305	0.582	0.177
`T*` text-sensitivity FA+GMM model (current text)	103	95.6	25	0.289	0.556	0.178
`T*` text-sensitivity FA model (legacy text)	103	103.0	25	0.281	0.545	0.178
`T*` text-sensitivity FA+GMM model (legacy text)	103	103.0	25	0.265	0.524	0.179

Note. `T*` is the 103-participant common set used to compare the two text representations. Current text denotes general sentence embeddings derived from participant utterances transcribed with gpt-4o-transcribe using the frozen `intfloat/multilingual-e5-base` encoder; legacy text denotes the segment embeddings derived from `bert_senti`. Target n is the number of participants in `T*`; mean evaluable participants per repeat is the mean number with estimated out-of-sample probabilities across repeats. All 25 repeats were retained, and each repeat-level metric used only rows with an estimated out-of-sample probability; complete prediction coverage for all 103 participants was not required. Of the 250 planned outer fits for the two current-text models (25 repeats $\times$ 5 outer folds $\times$ 2 models), 17 were not estimated: 8 for FA and 9 for FA+GMM. In 16 fits, no admissible Gaussian

mixture fit was found during current-text representation fitting; in one FA+GMM fit, logistic regression did not converge in an inner fold. This sensitivity analysis used the separate `cv_split_bank_text.csv` split bank. The openSMILE, HuBERT, and PyFeat factor scores were held fixed at their precomputed full-data values, whereas the current or legacy text factor and GMM were re-estimated within each inner and outer training split. The current-text runtime identifiers were `fa4_text_main` and `fagmm4_text_main` in run `initial_qc_v6`. By contrast, the primary current-text models reported in Table 2 of the main text and Table S5 used the primary split bank, re-estimated all four representations within the nested splits, and required complete participant predictions for a repeat. Thus, the AP values 0.305 and 0.289 here differ from the primary AP values 0.302 and 0.282 because the split bank, representation-fitting strategy, and evaluability rule differed; the values are not intended to reproduce the primary analysis. AP, average precision; FA, factor analysis; GMM, Gaussian mixture model; ROC-AUC, area under the receiver operating characteristic curve. Higher AP and ROC-AUC values and lower Brier scores are better. Differences were calculated from unrounded values and may not equal differences between the displayed rounded values.

Within each text representation, mean paired differences across repeats were calculated as the FA model minus the corresponding FA+GMM model. The differences were ΔAP +0.016, ΔROC-AUC +0.027, and ΔBrier −0.0002 for current text and ΔAP +0.016, ΔROC-AUC +0.021, and ΔBrier −0.0004 for legacy text.

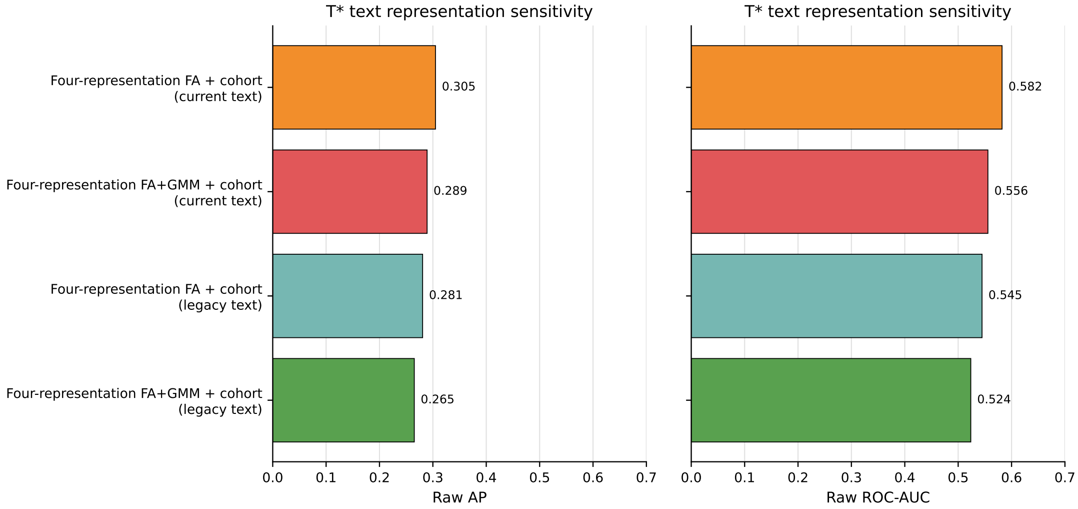

Figure S3. Raw average precision (AP) and area under the receiver operating characteristic curve (ROC-AUC) for the factor analysis (FA) and FA plus Gaussian mixture model (FA+GMM) models using the current and legacy text representations in `T*`.

S3d. Distributional Extension

Table S8. Participant-Level Metrics in the Exploratory Distributional Extension Using `DA*`

Model	Evaluable repeats	Raw AP	Raw ROC-AUC	Raw Brier score	Recalibrated AP	Recalibrated ROC-AUC	Recalibrated Brier score
Distributional FA	25	0.258	0.516	0.180	0.237	0.472	0.182
Distributional FA+GMM	25	0.248	0.492	0.180	0.239	0.478	0.183

Note. `DA*` is the 102-participant analysis set used for the exploratory distributional extension. AP, average precision; FA, factor analysis; GMM, Gaussian mixture model; ROC-AUC, area under the receiver operating

characteristic curve. Higher AP and ROC-AUC values and lower Brier scores are better. Differences were calculated from unrounded values and may not equal differences between the displayed rounded values.

The mean paired differences across repeats for distributional FA+GMM minus distributional FA were ΔAP −0.010, ΔROC-AUC −0.023, and ΔBrier −0.0001 for the raw metrics and ΔAP +0.002, ΔROC-AUC +0.006, and ΔBrier +0.0012 for the recalibrated metrics.

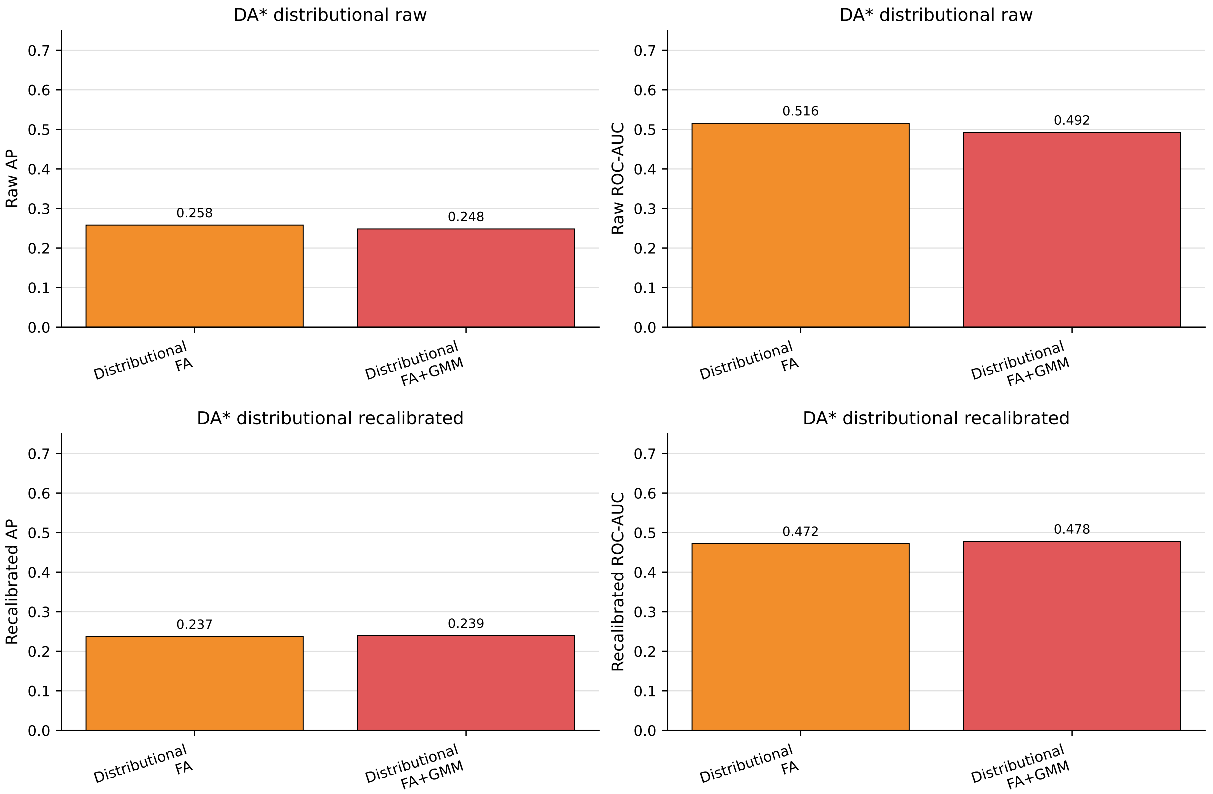

Figure S4. Raw and recalibrated average precision (AP) and area under the receiver operating characteristic curve (ROC-AUC) for the distributional factor analysis (FA) and FA plus Gaussian mixture model (FA+GMM) models in `DA*`.

S3e. Exploratory Length-Stability Sensitivity Analysis

Table S9. Performance Differences Calculated as the Prefix Model Minus the Same-Phase Full Model in the Exploratory Length-Stability Sensitivity Analysis

Phase	Prefix N	Target n	Mean paired n	Mean positive n	ΔAP	ΔROC-AUC	Raw ΔBrier score	Recalibrated ΔBrier score
`happy`	16	96	92.0	20.0	−0.120	−0.147	+0.003	−0.007
`happy`	32	67	67.0	13.0	−0.031	−0.054	+0.001	+0.003
`happy`	48	39	38.3	6.9	−0.020	−0.023	−0.005	−0.007
`sad`	16	96	90.4	19.8	−0.002	−0.012	−0.002	+0.002
`sad`	32	65	65.0	13.0	+0.062	+0.092	−0.002	+0.003
`sad`	48	48	45.0	10.3	+0.035	+0.053	−0.001	−0.026
`anger`	16	87	74.6	16.2	+0.013	−0.023	+0.005	+0.028
`anger`	32	59	56.7	13.5	+0.052	+0.060	−0.003	+0.008
`anger`	48	37	32.9	8.1	−0.002	−0.021	−0.000	−0.007
`lifestyle`	16	77	62.4	15.1	−0.060	−0.083	+0.110	−0.001
`lifestyle`	32	41	40.1	11.7	+0.003	+0.050	+0.095	+0.015

Phase	Prefix N	Target n	Mean paired n	Mean positive n	ΔAP	ΔROC-AUC	Raw ΔBrier score	Recalibrated ΔBrier score
`lifestyle`	48	21	16.3	3.9	−0.079	−0.006	+0.070	−0.044

Note. The gap was calculated as the prefix model minus the same-phase full model. Positive differences in AP and ROC-AUC and negative differences in the Brier score favor the prefix model. Target n denotes the number of participants in the deterministic subset; mean paired n and mean positive n are counts averaged across the 10 paired repeat subsets. All 10 repeats were retained, and each repeat-level comparison used only participants with estimated out-of-sample probabilities from both the prefix and same-phase full models in the corresponding cell. Of the 1,200 planned outer fits across the 12 phase–prefix-length cells and two models (10 repeats × 5 outer folds), 45 were not estimated: 34 prefix-model fits and 11 same-phase full-model fits. In 42 fits, no admissible Gaussian mixture fit was found; in 3 same-phase full-model fits, logistic regression did not converge in an inner fold. Because the prevalence-based no-skill AP baseline depends on the positive rate in each cell, AP gaps should not be directly compared across cells with different target n, mean paired n, and mean positive n. AP, average precision; ROC-AUC, area under the receiver operating characteristic curve.

Table S10. Correspondence Between Number of Segments and Participant Speech Duration in the Exploratory Length-Stability Sensitivity Analysis

Phase	Prefix N	Target n	Prefix speech duration, median [IQR]	Same-phase full speech duration, median [IQR]
happy	16	96	1:17 [1:01–1:46]	3:16 [2:42–4:27]
happy	32	67	2:11 [1:46–2:41]	3:52 [3:01–4:47]
happy	48	39	3:01 [2:42–3:50]	4:29 [3:44–5:08]
sad	16	96	1:21 [1:07–1:50]	3:29 [2:36–4:53]
sad	32	65	2:30 [1:58–2:55]	4:20 [3:06–5:27]
sad	48	48	3:29 [2:43–4:09]	4:45 [3:51–6:04]
anger	16	87	1:16 [1:00–1:47]	3:32 [2:26–4:37]
anger	32	59	2:20 [1:46–2:54]	4:06 [3:01–5:25]
anger	48	37	3:19 [2:46–4:04]	4:41 [3:57–6:00]
lifestyle	16	77	1:06 [0:49–1:21]	2:06 [1:33–3:05]
lifestyle	32	41	1:48 [1:35–2:17]	2:52 [2:06–3:52]
lifestyle	48	21	2:42 [2:18–3:56]	3:37 [3:09–4:20]

Note. Values are median participant speech durations (IQRs), expressed as minutes:seconds. The prefix model used the first 16, 32, or 48 complete four-representation segments in chronological order within each interview phase. The same-phase full model used all segments from the same interview phase for the participant set in the corresponding prefix-N analysis. Because segment lengths vary, the actual time corresponding to a given prefix length N differs by interview phase and participant. IQR, interquartile range.

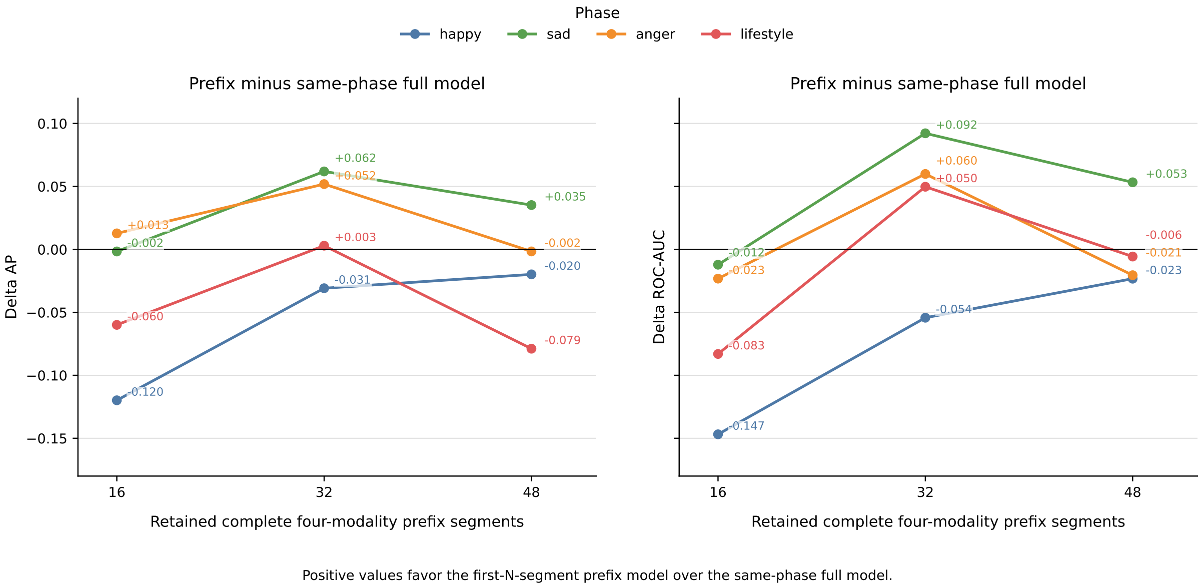

Figure S5. Raw average precision (AP) and area under the receiver operating characteristic curve (ROC-AUC) gaps in the exploratory length-stability sensitivity analysis. Differences are prefix minus same-phase full; positive AP and ROC-AUC values favor the prefix model.

S4. Fixed-Summary Overlap, Cross-Validation Diagnostics, and Analysis Provenance

To assess the extent to which GMM-derived posterior summaries overlapped with mean FA scores, we conducted exploratory diagnostics using precomputed fixed participant-level summaries. FA and GMM were not re-estimated for these diagnostics. For the overall and interview-phase-specific summaries, we calculated correlations between GMM summaries and mean FA scores, the proportion of variance explained when each GMM summary was regressed on the four mean FA scores, and univariate correlations between the resulting residuals and the PHQ-9 total score or screen-positive label. These exploratory descriptive diagnostics are distinct from the primary-analysis performance estimates and do not constitute a fold-specific predictive comparison. We also conducted an exploratory fixed-summary nested-CV sensitivity analysis using the primary split bank. In the residualized model, each GMM summary was regressed on the four mean FA summaries within each applicable inner or outer training fold, and the fitted relation was applied to the corresponding held-out rows. The downstream ridge logistic model and its penalty were refitted within the nested splits, but the underlying FA and GMM participant summaries remained precomputed fixed inputs. This section also documents the provenance of the phase-specific fixed-summary diagnostic and distinguishes it from the four-representation models reported in the main text and Tables S5, S7, and S14.

Table S11. Overall Fixed-Summary Diagnostics: Variance Explained by Mean FA Scores and Correlations with PHQ-9 Outcomes

GMM summary	n	R^2 from mean FA scores	Residual variance proportion	Raw r (PHQ-9 total)	Raw r (screen-positive)	Residual r (PHQ-9 total)	Residual r (screen-positive)
Soft component-2 occupancy summary	103	0.763	0.237	0.016	−0.008	0.117	0.152
Posterior assignment-margin summary	103	0.664	0.336	0.054	0.050	0.005	−0.062

Note. FA, factor analysis; GMM, Gaussian mixture model; PHQ-9, Patient Health Questionnaire-9. R^2 is the coefficient of determination when each GMM summary is linearly regressed on the four participant-level mean FA scores for openSMILE, HuBERT, PyFeat, and text. The residual variance proportion is $1 - R^2$. Here, r denotes Pearson's correlation coefficient, and `screen-positive` is the binary indicator for a PHQ-9 total score of 10 or higher.

Table S12. Pearson Correlations of Interview-Phase-Specific Mean FA Scores and GMM Summaries with PHQ-9 Total Scores

Interview phase	openSMILE factor	HuBERT factor	PyFeat factor	Text factor	Soft component-2 occupancy summary	Posterior assignment-margin summary
Happy	−0.055	0.046	−0.265	−0.112	0.086	−0.002
Sad	−0.076	0.037	−0.279	−0.110	0.086	0.000
Anger	−0.011	0.023	−0.228	0.039	0.012	−0.033
Lifestyle	−0.151	0.024	−0.235	0.002	−0.160	0.146

Note. Values are Pearson correlation coefficients. FA, factor analysis; GMM, Gaussian mixture model; PHQ-9, Patient Health Questionnaire-9.

Table S13. Pearson Correlations of Interview-Phase-Specific Mean FA Scores and GMM Summaries with the PHQ-9 Screen-Positive Label

Interview phase	openSMILE factor	HuBERT factor	PyFeat factor	Text factor	Soft component-2 occupancy summary	Posterior assignment-margin summary
Happy	−0.086	−0.013	−0.223	−0.180	0.037	0.011
Sad	0.003	−0.004	−0.215	−0.132	0.124	−0.056
Anger	−0.052	−0.054	−0.187	0.025	−0.035	−0.004
Lifestyle	−0.184	−0.019	−0.235	0.061	−0.188	0.167

Note. Values are Pearson correlation coefficients. FA, factor analysis; GMM, Gaussian mixture model; PHQ-9, Patient Health Questionnaire-9.

Table S14. Fixed-Summary Residualized-GMM Sensitivity and Provenance of Related Cross-Validation Results

Panel A. Residualized-GMM sensitivity based on fixed summaries

Model	Evaluable repeats	AP	ROC-AUC	Brier score
FA model based on fixed summaries (`fa4_text_fixed`)	25	0.309	0.593	0.1771
FA+GMM model based on raw fixed summaries (`fagmm4_text_fixed`)	25	0.284	0.553	0.1788
FA+GMM model with fold-wise residualized fixed GMM summaries (`fagmm4_text_residualized_gmm_fixed`)	25	0.298	0.561	0.1786

Note. AP, average precision; FA, factor analysis; GMM, Gaussian mixture model; ROC-AUC, area under the receiver operating characteristic curve. Values are means across evaluable repeats. The mean paired differences across repeats for raw fixed-summary FA+GMM minus fixed-summary FA were ΔAP −0.026, ΔROC-AUC −0.040, and ΔBrier +0.0017. The corresponding differences for residualized FA+GMM minus fixed-summary FA were ΔAP −0.011, ΔROC-AUC −0.032, and ΔBrier +0.0015. Residualized FA+GMM minus raw fixed-summary FA+GMM yielded ΔAP +0.014, ΔROC-AUC +0.008, and ΔBrier −0.0002. Residualization was estimated within each applicable inner or outer training fold, whereas the underlying participant-level FA and GMM summaries were precomputed fixed inputs. Panel A is therefore an exploratory downstream-model diagnostic rather than a full-pipeline internal-validation comparison. Higher AP and ROC-AUC values and lower Brier scores are better.

Panel B. Analysis provenance

Result location and model	Analysis set and split bank	Representation and fitting	Missingness and evaluability rule	Repeats and run identifier
Table 2 of the main text and Table S5: primary four-representation FA (`fa4_text`)	`S*`; 103 participants; `cv_split_bank_primary.csv`	Current E5 text; all four FA representations re-estimated within inner and outer training splits	A repeat required predictions for all 103 participants	25 repeats; `initial_qc_v6_step09_rerun`
Table 2 of the main text and Table S5: primary four-representation FA+GMM (`fagmm4_text`)	`S*`; 103 participants; `cv_split_bank_primary.csv`	Current E5 text; all four FA representations and the GMM re-estimated within inner and outer training splits	A repeat required predictions for all 103 participants	24 repeats; one method failure; `initial_qc_v6_step09_rerun`

Result location and model	Analysis set and split bank	Representation and fitting	Missingness and evaluability rule	Repeats and run identifier
Table S5: two-factor-per-modality FA (fa4_text_cap2)	S*; 103 participants; cv_split_bank_primary.csv	Current E5 text; a fixed two-factor capacity for each modality re-estimated within inner and outer training splits, with a zero-filled second factor when only one was estimable	A repeat required predictions for all 103 participants	25 repeats; initial_qc_v6_step09_rerun
Table S5: two-factor-per-modality FA+GMM (fagmm4_text_cap2)	S*; 103 participants; cv_split_bank_primary.csv	The same fold-specific two-factor representations plus a two-component GMM re-estimated within each inner and outer training split	A repeat required predictions for all 103 participants	25 repeats; initial_qc_v6_step09_rerun
Table S5: three-component-GMM FA+GMM (fagmm4_text_k3)	S*; 103 participants; cv_split_bank_primary.csv	Current E5 text; one-factor representations and a three-component GMM re-estimated within inner and outer training splits; ordered component-2 and component-3 posterior means and the assignment margin were summarized	A repeat required predictions for all 103 participants	25 repeats; initial_qc_v6_step09_rerun
Table 5 of the main text: phase-specific fixed-summary FA and FA+GMM (fa3, fagmm3)	Phase-specific analyzable sets; 103 participants for happy and sad, 102 for anger and lifestyle; cv_split_bank_topic.csv	Precomputed duration-weighted three-representation FA and GMM summaries; downstream logistic model refitted within nested splits	Each repeat required predictions for all participants in the corresponding phase	25 repeats for every model–phase cell; FS-20260821
Table S7: T* text-sensitivity FA, current text (fa4_text_main)	T*; 103 participants; separate cv_split_bank_text.csv	Non-text factor scores fixed at precomputed full-data values; current E5 text factor re-estimated within inner and outer training splits	Each repeat used available rows with estimated predictions; complete coverage was not required	25 repeats; mean evaluable $n = 96.4$; initial_qc_v6
Table S7: T* text-sensitivity FA+GMM, current text (fagmm4_text_main)	T*; 103 participants; separate cv_split_bank_text.csv	Non-text factor scores fixed at precomputed full-data values; current E5 text factor and GMM re-estimated within inner and outer training splits	Each repeat used available rows with estimated predictions; complete coverage was not required	25 repeats; mean evaluable $n = 95.6$; initial_qc_v6
Table S14, Panel A: overall fixed-summary FA and raw FA+GMM (fa4_text_fixed, fagmm4_text_fixed)	S*; 103 participants; cv_split_bank_primary.csv	Precomputed full-data four-representation FA and GMM summaries; downstream logistic model refitted within nested splits	Each repeat required predictions for all 103 participants	25 repeats for each model; FS-20260821
Table S14, Panel A: residualized fixed-summary FA+GMM (fagmm4_text_residualized_gmm_fixed)	S*; 103 participants; cv_split_bank_primary.csv	Same precomputed summaries; GMM residualization and downstream logistic model refitted within each applicable inner or outer training fold	Each repeat required predictions for all 103 participants	25 repeats; FS-20260821

Note. The primary rows in Table S5 are repeated outputs from the same Step09 run used for Tables 2 and 4 of the main text, so their raw AP values are identical by design (0.302 for FA and 0.282 for FA+GMM). Table S7 reports a distinct text-sensitivity pipeline. Its current-text AP values (0.305 for FA and 0.289 for FA+GMM) differ because it used a separate split bank, fixed the three non-text representations, and scored each repeat using available estimated predictions rather than requiring complete participant coverage. The differences are therefore expected consequences of analysis design and are not rounding differences or an attempted reproduction of the primary estimates. The cap2 and K=3 rows in Table S5 were generated by the Step12 fold-specific sensitivity pipeline using the same primary split bank. The K=3 FA comparator is the primary fa4_text output because the sensitivity changed only the number of GMM components. FS-20260821 denotes the phase-specific and overall fixed-summary analyses based on precomputed inputs from initial_qc_v6. For these fixed-summary fits, the ridge mapping was $C = 1/(\lambda n_{\text{train}})$. All design columns were centered only for solver stability and the intercept was transformed back to the original design coordinates; this numerical parameterization did not change the predictions or estimand.

S5. Reproducibility Details for Feature Extraction and Representation Construction

This section summarizes the feature-block structure and GMM fit diagnostics using the analysis input tables and cross-validation outputs.

S5a. Feature-Block Structure and GMM Fit Diagnostics

Table S15. Input Structures and Dimensions of Feature Blocks

Feature block	Segment input structure	Specification	Dimensions or number of columns
openSMILE	`id`, start time, end time, vector	GeMAPSv01a feature set; FeatureID 0–61	62
HuBERT	`id`, start time, end time, segment vector	Precomputed HuBERT vectors at the speech-segment level	768
PyFeat	Frame-level output aggregated to speech-segment level	Frames with FaceScore $\geq$ 0.90 were treated as valid; segments with fewer than 5 valid frames were treated as missing.	30
Text	Sentence embedding of participant speech	`intfloat/multilingual-e5-base`, `passage:` prefix, attention-mask mean pooling, L_2 normalization, maximum sequence length 512	768

Note. HuBERT, Hidden-Unit BERT; L_2, Euclidean norm; PyFeat, Python Facial Expression Analysis Toolbox.

Table S16. PyFeat Column Structure and Segment Summaries Retained for Analysis

Category	Columns or retained summaries
Raw frame-level output	FaceScore; x- and y-coordinates of 68 landmarks; pitch, roll, and yaw; AU01, AU02, AU04, AU05, AU06, AU07, AU09, AU10, AU11, AU12, AU14, AU15, AU17, AU20, AU23, AU24, AU25, AU26, AU28, and AU43; anger, disgust, fear, happiness, sadness, surprise, and neutral; Identity and Identity_1–Identity_512
Head-pose summaries retained for analysis	`pitch_mean`, `pitch_sd`, `roll_mean`, `roll_sd`, `yaw_mean`, `yaw_sd`
Action Unit summaries retained for analysis	Means and standard deviations of AU01, AU04, AU06, AU12, AU15, AU17, AU20, AU25, and AU26
Expression-probability summaries retained for analysis	Means and standard deviations of happiness, sadness, and neutral

Category	Columns or retained summaries
Valid-frame rate	Among 17,522 segments with available PyFeat data: mean 0.986, median 1.000, IQR 1.000–1.000, range 0.000–1.000. Among 17,164 segments meeting the analytic usability criteria: mean 0.991, median 1.000, IQR 1.000–1.000, range 0.200–1.000.

Note. AU, facial action unit; IQR, interquartile range; PyFeat, Python Facial Expression Analysis Toolbox.

Table S17. Retained Feature Counts in Primary-Analysis Outer Fits

Model	openSMILE	HuBERT	PyFeat	Text	Number of participant-level input variables
FA model	62/62	768/768	30/30	768/768	5
FA+GMM model	62/62	768/768	30/30	768/768	7
Three-representation FA model	62/62	768/768	30/30	–	4
Three-representation FA+GMM model	62/62	768/768	30/30	–	6

Note. Values are shown as retained/raw feature counts. No successful outer fit excluded any features because of zero variance. FA, factor analysis; GMM, Gaussian mixture model.

Table S18. Primary-Analysis GMM Fit Diagnostics

Model	Outer fits included	Selected covariance regularization	Convergence and acceptance status	Range of minimum component occupancy	Range of the minimum number of participants per component
FA+GMM model	124	10^{-5} in all fits	The initial GMM candidate converged and satisfied the occupancy criteria in all fits	0.0527–0.4479	75–84
Three-representation FA+GMM model	125	10^{-5} in all fits	The initial GMM candidate converged and satisfied the occupancy criteria in all fits	0.0558–0.4144	71–84

Note. For the primary FA+GMM model, 1 of 125 outer fits failed because logistic regression remained non-convergent at $\lambda = 1$ in inner fold 1 of repeat 8, outer fold 2, after the single retry specified in Section 14.1 of the statistical analysis plan. Before the retry, all input values were finite and no zero-variance or near-constant columns were detected. The iteration limit was increased from 10,000 to 20,000, retaining the same design matrix, outcomes, split, penalty, solver, and tolerance (10^{-8}). Both attempts terminated abnormally after nine optimizer iterations, before reaching either iteration limit. The 21 test-participant predictions remained `not_estimated`. Thus, 124 successful outer fits were summarized for GMM diagnostics, and the primary FA+GMM performance summary and its paired comparison with FA used 24 complete repeats. The failure did not result from unmet GMM occupancy criteria or a change in covariance regularization. FA, factor analysis; GMM, Gaussian mixture model.

S5b. Supplementary Transcript and Segment Availability Checks

Table S19. Supplementary Transcript and Segment Availability Checks

All 103 participants in the common set for the primary analysis passed the structural checks for transcript availability, and participant-level openSMILE, HuBERT, PyFeat, text, and GMM summaries were successfully computed for every participant. The following summaries are descriptive and were obtained after deterministic QC. These checks assessed structural availability and processing completeness; they did not assess transcription accuracy against reference transcripts. For performance estimation, preprocessing, FA, GMM fitting, and participant-level aggregation were repeated separately within each split.

Stratum	*n*	Complete four-representation segments, median [IQR]	Total duration of complete segments (ms), median [IQR]	PyFeat exact-join usability rate, median [IQR]	Passed structural transcript-availability checks, *n*/*N*	Exact-join coverage <95%, *n*
Screen-negative	79	155 [91–218]	747619 [592170–908590]	0.991 [0.974–1.000]	79/79	0
Screen-positive	24	148 [82–270]	757111 [549545–1075470]	0.991 [0.981–1.000]	24/24	0
Adult stratum	83	155 [86–240]	750500 [560300–936620]	0.992 [0.979–1.000]	83/83	0
Pediatric stratum	20	154 [127–226]	691570 [640074–898566]	0.985 [0.970–0.994]	20/20	0

Note. GMM, Gaussian mixture model; IQR, interquartile range; ms, milliseconds; PyFeat, Python Facial Expression Analysis Toolbox; QC, quality control.

The numbers of participants meeting the strict analyzability criteria in each interview phase were 103 for happy (24 screen-positive), 103 for sad (24 screen-positive), 102 for anger (24 screen-positive), and 102 for lifestyle (24 screen-positive). The median numbers (IQRs) of complete three-representation segments in each phase were 43 [24–56] for happy, 43 [26–61] for sad, 36 [23–63] for anger, and 28 [16–39] for lifestyle. No text-encoder failures occurred during text QC. Segments that contained only whitespace or nonverbal tags were treated as missing sentence embeddings according to the predefined rules.

S6. Post Hoc Sensitivity Analyses

S6a. Adult-Only Cohort-Composition Sensitivity

The two-group cohort indicator used in the design-only benchmark for the primary analysis was a coarse design variable that may capture differences in recruitment pathway, clinical background, and screen-positive prevalence between the adult and pediatric strata. Therefore, as an additional post hoc sensitivity analysis, we restricted the sample to the adult-only analysis set `A*` within the common set for the primary analysis and conducted repeated nested CV while retaining the distinction between university students and adult patients as the within-adult source variable. In this analysis, we compared three models: the adult-source design-only benchmark; the adult-source FA model (runtime ID `adult_fa4_text`), which included the source indicator and one mean FA score from each of openSMILE, HuBERT, PyFeat, and text; and the adult-source FA+GMM model (runtime ID `adult_fagmm4_text`), which added only the two GMM-derived posterior summaries to the preceding FA model.

Table S20. Composition of the Adult-Only Analysis Set `A*`

Group	n	Screen-positive	Screen-negative	Screen-positive rate
Adult-only analysis set `A*` overall	83	15	68	18.1%
University students	34	6	28	17.6%
Adult patients	49	9	40	18.4%

Note. `A*` denotes the 83-participant adult-only analysis set used in the post hoc sensitivity analysis.

Table S21. Participant-Level Metrics for Adult-Only Cohort-Composition Sensitivity

Model or comparison	Evaluable repeats	AP	ROC-AUC	Brier score
Adult-source design-only benchmark	25	0.164	0.463	0.149
Adult-source FA model	25	0.212	0.509	0.148
Adult-source FA+GMM model	25	0.197	0.483	0.149
Adult-source FA model minus adult-source design-only benchmark	25	+0.051	+0.037	−0.00002
Adult-source FA+GMM model minus adult-source FA model	25	−0.016	−0.026	+0.00015

Note. The adult-source design-only benchmark (runtime ID `adult_reference`) uses only the source variable indicating whether a participant was a university student or an adult patient within the adult-only analysis set. AP denotes average precision; FA, factor analysis; GMM, Gaussian mixture model; ROC-AUC, area under the receiver operating characteristic curve. Higher AP and ROC-AUC are better, and lower Brier scores are better. In the difference rows, positive AP and ROC-AUC differences favor the model on the left, and a negative difference in Brier score favors the model on the left. This analysis is a post hoc sensitivity analysis restricted to the adult stratum and does not replace the primary-analysis estimand, which includes adult and pediatric participants.

S6b. GMM-Input Scaling Sensitivity

The prespecified FA+GMM model entered the four segment-level FA factor scores into each GMM on their fold-specific native scales. Because the median openSMILE factor-score standard deviation was substantially larger than those of HuBERT, PyFeat, and text, we conducted a post hoc scale-handling sensitivity analysis to determine whether the performance of the GMM-augmented model depended on this implementation choice. This analysis retained the same 103 participants, outcome labels, participant-level split bank, seed assignments, fold-specific FA representations, GMM settings and acceptance criteria, participant-level aggregation, ridge logistic regression, tuning grid, and scoring procedures used in the primary analysis.

For each outer and inner GMM fit, the four factor-score coordinates were centered and divided by population standard deviations estimated only from the participant-balanced GMM training segments. The resulting training means and standard deviations were then applied without re-estimation to the corresponding validation or test segments. Thus, the outer-test data did not contribute to outer-fold standardization, and the inner-validation data did not contribute to inner-fold standardization. The scale-handling contrast was the standardized-input FA+GMM model minus the native-scale FA+GMM model, and the incremental-value contrast under the alternative scaling specification was the standardized-input FA+GMM model minus the FA model. Raw AP was the primary metric; raw ROC-AUC, raw Brier score, and recalibrated Brier score were supporting metrics.

Reproducibility checks matched all 125 outer-fold and 500 inner-fold FA manifests, confirmed training-only standardization for all 625 GMM fits, and matched all 2,575 participant-level out-of-fold prediction keys to the split bank used in the primary analysis. The standardized-input FA+GMM model had no fitting failures and contributed all 25 repeats. The native-scale FA+GMM model contributed 24 repeats because its previously reported failure remained classified as `not_estimated`.

Table S22. Mean Participant-Level Metrics Across Evaluable Repeats in the Post Hoc GMM-Input Scaling Sensitivity Analysis

Model	Evaluable repeats	Raw AP	Raw ROC-AUC	Raw Brier score	Recalibrated AP	Recalibrated ROC-AUC	Recalibrated Brier score
FA model	25	0.302	0.576	0.1772	0.279	0.540	0.1823
FA+GMM model with native-scale GMM inputs	24	0.282	0.546	0.1788	0.263	0.515	0.1847
FA+GMM model with standardized GMM inputs	25	0.300	0.565	0.1771	0.275	0.529	0.1856

Note. AP, average precision; FA, factor analysis; GMM, Gaussian mixture model; ROC-AUC, area under the receiver operating characteristic curve. Values are means across model-evaluable repeats. Higher AP and ROC-AUC and lower Brier scores are better. Recalibrated AP and ROC-AUC are auxiliary descriptive values because recalibration used different cross-fitted transformations across outer folds.

Table S23. Paired Differences in the Post Hoc GMM-Input Scaling Sensitivity Analysis

Comparison	Metric	Common repeats	Mean difference across repeats	Median difference [IQR]	Favorable repeats	Within-participant mean difference (95% conditional interval)
Standardized-input FA+GMM model minus native-scale FA+GMM model	Raw AP	24	+0.0179	+0.0176 [+0.0081 to +0.0273]	21/24	+0.0496 [+0.0070 to +0.1304]
Standardized-input FA+GMM model minus native-scale FA+GMM model	Raw ROC-AUC	24	+0.0202	+0.0216 [+0.0008 to +0.0372]	18/24	+0.0348 [−0.0063 to +0.0754]
Standardized-input FA+GMM model minus native-scale FA+GMM model	Raw Brier score	24	−0.00172	−0.00105 [−0.00244 to +0.00008]	16/24	−0.00225 [−0.00428 to −0.00043]
Standardized-input FA+GMM model minus native-scale FA+GMM model	Recalibrated Brier score	24	+0.00091	−0.00137 [−0.00691 to +0.00133]	15/24	Not calculated
Standardized-input FA+GMM model minus FA model	Raw AP	25	−0.0014	−0.0075 [−0.0187 to +0.0147]	11/25	+0.0022 [−0.0524 to +0.0588]
Standardized-input FA+GMM model minus FA model	Raw ROC-AUC	25	−0.0103	−0.0127 [−0.0280 to +0.0063]	8/25	−0.0095 [−0.0522 to +0.0332]
Standardized-input FA+GMM model minus FA model	Raw Brier score	25	−0.00010	+0.00001 [−0.00102 to +0.00067]	12/25	−0.00072 [−0.00216 to +0.00064]
Standardized-input FA+GMM model minus FA model	Recalibrated Brier score	25	+0.00335	+0.00042 [−0.00229 to +0.00593]	11/25	Not calculated

Note. AP, average precision; FA, factor analysis; GMM, Gaussian mixture model; IQR, interquartile range; ROC-AUC, area under the receiver operating characteristic curve. Each difference was calculated as the model named first minus the comparator named second. For AP and ROC-AUC, a positive difference is favorable; for Brier score, a negative difference is favorable. The favorable-repeat count is the number of paired repeats with differences in the favorable direction. Repeat summaries describe split-induced variability in repeated internal validation and do not represent independent external validation sets. Conditional intervals were calculated from 2,000 outcome-stratified participant-level bootstrap resamples, using each participant's mean out-of-fold probability across the common evaluable repeats. They are conditional on the fixed split bank and generated predictions and do not propagate uncertainty from refitting the complete pipeline. No conditional interval was calculated for differences in recalibrated Brier score.

Standardizing the GMM inputs yielded more favorable mean raw AP and ROC-AUC values and a lower mean raw Brier score across repeats than the native-scale FA+GMM model, although the mean difference in recalibrated Brier score did not favor standardization. Relative to the FA model, however, the standardized-input FA+GMM model had nearly identical mean raw AP and Brier scores and a lower mean raw ROC-AUC across repeats; none of the three within-participant conditional intervals for the raw metrics excluded zero. The sensitivity analysis therefore showed that the evaluated GMM pipeline was sensitive to input scaling, but standardization did not produce a clear incremental performance benefit beyond the FA model.

For descriptive context on the probability scale, Figure S6 shows the distributions of participant-level

mean estimated probabilities underlying the three primary model summaries. Panel A presents the full probability range, whereas Panel B repeats the same observations on an explicitly labeled zoomed scale so that distributional overlap can be inspected without implying a larger absolute separation.

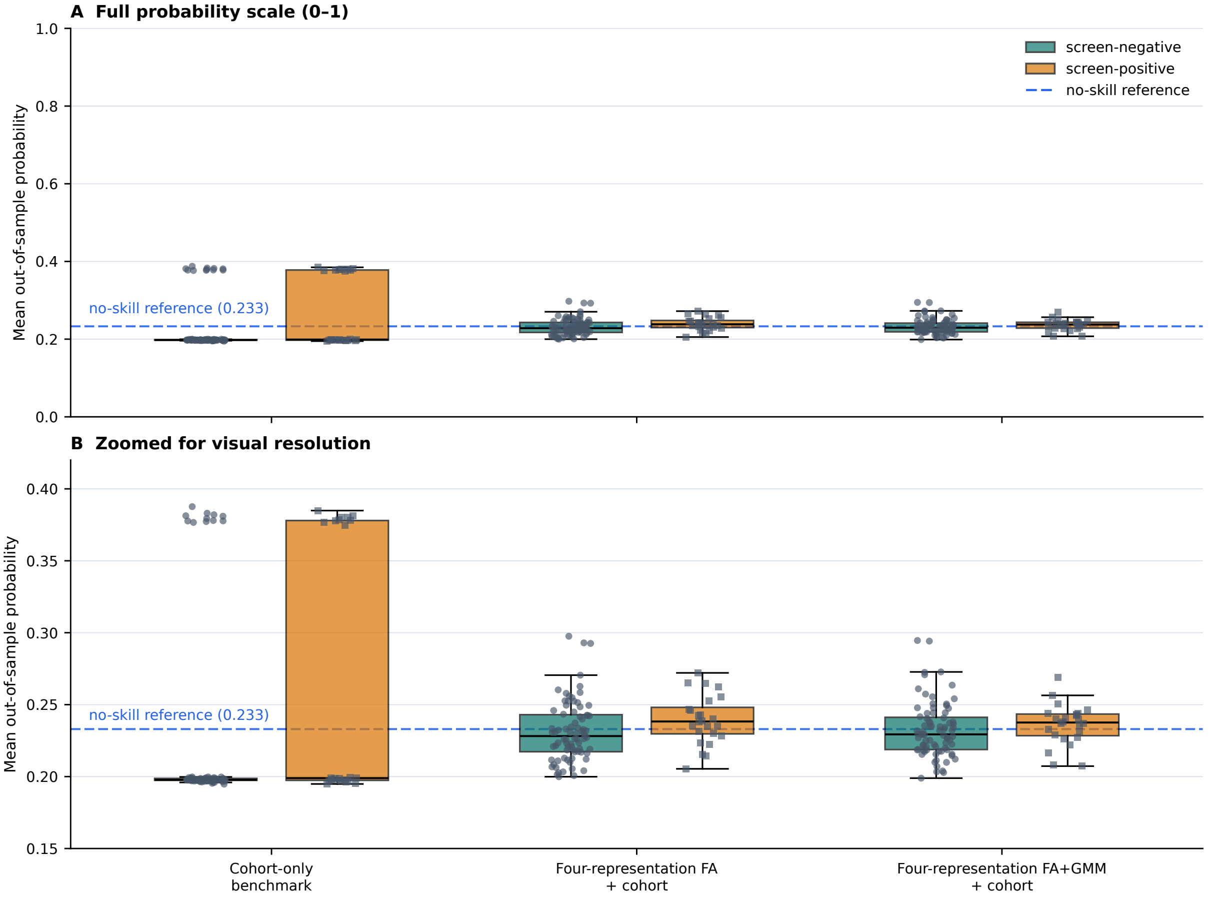

Figure S6. Distributions of out-of-sample estimated probabilities averaged across repeats for each participant, stratified by J-PHQ-9 screen-positive status, for the three primary models. Panel A displays the full 0–1 probability range. The y-axis in Panel B is zoomed for visual resolution. The panels show identical observations and differ only in y-axis scale. The dashed horizontal line denotes the no-skill reference corresponding to the overall screen-positive rate ($24/103 = 0.233$). Participant-level means were calculated across 25 complete repeats for the design-only and FA models and across 24 complete repeats for the FA+GMM model; the incomplete FA+GMM repeat was excluded in full. Abbreviations: FA, factor analysis; GMM, Gaussian mixture model; J-PHQ-9, Japanese version of the Patient Health Questionnaire-9.

S7. Abbreviations

Abbreviation, symbol, or model name	Definition
A*	Adult-only analysis set used in the adult-only post hoc sensitivity analysis.
AP	Average precision.
AU	Facial Action Unit.
CV	Cross-validation.
DA*	Exploratory distributional-extension analysis set.
FA	Factor analysis.
FA+GMM	Model or feature set that adds GMM-derived posterior summaries to mean FA scores.
GeMAPSv01a	Geneva Minimalistic Acoustic Parameter Set, version 01a.
GMM	Gaussian mixture model.
gpt-4o-transcribe	OpenAI transcription model used for transcript generation.
HuBERT	Hidden-Unit BERT speech representation model.
ID	Identifier.
IQR	Interquartile range.
J-PHQ-9	Japanese version of the Patient Health Questionnaire-9.
K	Number of GMM components.
L_2	Euclidean norm used for vector normalization and coefficient penalization.
MINRES	Minimum residual factor-analysis extraction method.
ms	Milliseconds.
N	Number of retained complete four-representation prefix segments in the length-stability sensitivity analysis, unless otherwise noted.
NFKC	Unicode Normalization Form Compatibility Composition.
openSMILE	Open-source Speech and Music Interpretation by Large-space Extraction, an acoustic-feature extraction toolkit.
PA*	Phase-aware prespecified secondary analysis set.
PCA	Principal component analysis.
PHQ-9	Patient Health Questionnaire-9.
PHQ-A	Patient Health Questionnaire for Adolescents.
PR-AUC	Area under the precision-recall curve.
PyFeat	Facial-feature representation derived using the Python Facial Expression Analysis Toolbox.
QC	Quality control.
R^2	Coefficient of determination.
ROC-AUC	Area under the receiver operating characteristic curve.
SD	Standard deviation.
S*	103-participant common analysis set used for the primary, supporting, and selected sensitivity analyses.
T*	Text representation sensitivity analysis set.

S8. Comparison with Previous Reports from the Interview Corpus

Table S24. Study Objectives, Representations, Validation, and Selected Results in Overlapping Samples

Study, sample, and objective	Representations and models	Validation / evaluation	Selected reported results
Kashihara et al. [3]; 25 university students. Corpus construction and exploratory feature–questionnaire associations.	GiNZA lexical features, openSMILE acoustic features, and OpenFace facial action units; correlation analysis.	Descriptive correlations in the collected sample; no held-out participant-level screening classifier evaluated.	The proportion of negative phrases correlated positively with PHQ-9 scores ($r > 0.5$).
Kiuchi et al. [4]; 99 participants. Four-class segment-level emotion/topic classification.	OpenFace, openSMILE, and VGGish; multifactor FA and a 15-component GMM, with demographic and questionnaire covariates; logistic regression, decision tree, random forest, and XGBoost.	Participant-disjoint training/validation/test sets (64%/16%/20%); fivefold StratifiedKFold for tuning. This was not the present participant-level PHQ-9 outcome comparison.	XGBoost test accuracy 0.3199 and balanced accuracy 0.2837 (four classes).
Matsumoto et al. [5]; 74 participants, including 13 with positive PHQ-9-derived labels. Dynamic fusion and affective pretraining.	SigLIP, head pose, acoustic and text features; concatenation, summation, gating, and cross-attention across fusion stages; 512 configurations.	Participant-independent CV; early stopping based on validation ROC-AUC. The report does not specify the fold count.	Audio-stage cross-attention had mean ROC-AUC 0.774 and PR-AUC 0.606 across the corresponding configurations.
Present study; 103 participants, including 24 screen-positive. Incremental value of GMM summaries beyond FA means and probability calibration.	openSMILE, HuBERT, PyFeat, and text embeddings; one FA factor per representation; ridge logistic regression with cohort indicator, with or without two GMM posterior summaries; design-only benchmark.	Preregistered participant-level nested CV (25 repeats, five outer and four inner folds); representations refitted within training splits; matched model comparisons and separate calibration diagnostics.	Mean AP: design-only 0.332, FA 0.302, native-scale FA+GMM 0.282. Post hoc standardized-input FA+GMM AP: 0.300.

Note. All three earlier samples are nested within the present 103-participant cohort and overlap with one another; the sample sizes must not be added. Earlier-study results summarize their respective reports, not a new reanalysis or a reproduction of their original tables. Tasks, participants, predictor sets, metrics, and evaluation procedures differ, so the values do not establish relative performance across studies. AP, average precision; CV, cross-validation; FA, factor analysis; GMM, Gaussian mixture model; PHQ-9, Patient Health Questionnaire-9; PR-AUC, area under the precision-recall curve; ROC-AUC, area under the receiver operating characteristic curve.

S9. Stored Factor Diagnostics across Outer Training Folds

Table S25. Descriptive Variation in Stored One-Factor Diagnostics

Representation	Features	Median |loading|	Maximum |loading|	Score SD
openSMILE	62	0.288 [0.235–0.316]	0.859 [0.843–0.878]	35.309 [23.346–50.555]
HuBERT	768	0.253 [0.240–0.266]	0.781 [0.757–0.826]	1.003 [1.001–1.004]
PyFeat	30	0.260 [0.217–0.292]	0.815 [0.793–0.845]	0.960 [0.957–0.963]
Text	768	0.276 [0.271–0.282]	0.791 [0.783–0.803]	1.032 [1.029–1.038]

Note. Values are the median [minimum–maximum] across the 125 outer training fits (25 repeats × five folds) for the primary four-representation factor analysis (FA) model. Features denotes the retained feature count, which was constant across these fits. Median and maximum absolute loadings were first summarized within each fitted representation and then across fits. Score SD is the standard deviation of factor scores in the segments used for that training fit; it is not the SD of participant-level means. These are dependent fits from overlapping training samples, and the ranges are descriptive, not confidence intervals. The model-estimation procedure and predictions were not changed for this diagnostic summary.

The saved manifests contain loading maxima and medians, feature names, sign-fixing metadata, and factor-score moments. They do not contain full loading vectors or the fitted correlation matrices needed to reconstruct explained variance, communalities, residual-correlation diagnostics, or featurewise loading stability. Variation in the aggregate loading summaries therefore does not establish stability of individual loadings or adequacy of the one-factor specification. Together with the existing two-factor sensitivity analysis (Table S5), these diagnostics characterize the evaluated representation and its scale without asserting that one factor captures all relevant information.

S10. Post Hoc Precision and Evaluation Sample-Size Sensitivity

Estimands and precision in the observed cohort

The primary precision estimand was the mean paired average-precision (AP) difference between the native-scale FA+GMM and FA models across their 24 common evaluable repeats. This differs from calculating AP after averaging each participant's probabilities. ROC-AUC and Brier differences used the same repeat-level aggregation. For calibration, the estimands were the mean joint intercept and slope across each model's evaluable repeats, as in Table 4 (25 for FA and 24 for FA+GMM), separately before and after recalibration.

We sampled 103 participants with replacement 2,000 times using seed 20260919, retaining each participant's outcomes and complete vector of predictions across all models and repeats. Each metric was recomputed within each repeat before averaging. The 2.5th and 97.5th percentiles gave conditional intervals for the repeat-mean contrasts. The calibration resamples and assessment regressions were identical to those used for Table 4, and the resulting calibration intervals were checked against that table. This ordinary bootstrap allowed the number of positive outcomes to vary; it differs from the outcome-stratified bootstrap of participant-mean predictions in Tables 2 and 3, Panel B.

Working population and sample-size calculation

Precision-based planning requires assumptions specific to the performance measure and evaluation population [6]. We used the empirical joint distribution of the outcome and the complete prediction vector as a working population. For an assumed positive proportion π, the mass assigned to participant i was

$$q_i(\pi) = \begin{cases} \pi/24, & y_i = 1, \\ (1-\pi)/79, & y_i = 0. \end{cases}$$

Thus, changing prevalence retained the class-conditional score distributions and the pairing of models and repeats. It did not simulate a specific change in the adult/pediatric composition. We examined $\pi = 0.15$, 24/103, and 0.30 and total sample sizes $N = 103$, 200, 400, 800, 1,600, 3,200, 6,400, 12,800, and 25,600. All targets and simulation settings were fixed before running this additional analysis; they were not part of the original preregistration.

For each scenario, we generated 2,000 independent evaluation samples using multinomial counts with size N and cell probabilities $q_i(\pi)$ (seed 20260920, with separate deterministic streams by scenario). Integer counts exactly represented duplicated draws without treating repeats as independent participants. At $N = 103$ and $\pi = 24/103$, the existing bootstrap draws were reused. Weighted AP retained tied scores at the same threshold and used the same noninterpolated definition as the original scoring. Calibration assessment regressions retained the original joint intercept/slope specification and numerical penalty. All draws were retained; single-class samples, nonidentifiability, or failed fits would stop the analysis rather than trigger an alternative estimator.

For each estimand, we estimated its sampling standard deviation s_N across the 2,000 samples and calculated the approximate normal-theory 95% interval full width

$$W_N = 2\Phi^{-1}(0.975)s_N.$$

The targets were full widths of 0.10, 0.05, and 0.02 for the AP difference and 0.80, 0.40, and 0.20 for a calibration slope. These represent alternative precision goals, not clinical acceptability thresholds or equivalence margins. We reported the first tested sample size with W_N at or below each target, together with the preceding tested size. This grid search does not identify an exact continuous minimum. Monte Carlo standard errors for W_N were estimated from the empirical fourth moment using the delta method. The sampling-distribution percentiles were also stored, but were not treated as confidence limits for a newly observed clinical cohort. Interval coverage and the probability that an individual future study would attain its target width were not evaluated.

Results

All 27 scenarios and 54,000 simulated samples completed. Table S26 reports the precision of the observed-cohort estimates. The primary repeat-mean AP difference had a conditional interval spanning zero. The broad calibration slope intervals corresponded to substantially larger projected evaluation samples when narrow slope intervals were targeted (Table S27).

Table S26. Conditional Precision of Repeat-Mean Estimates at $N = 103$

Estimand	Estimate	Conditional 95% interval	Full width
ΔAP	-0.020	[-0.046, 0.001]	0.048
ΔROC-AUC	-0.031	[-0.053, -0.008]	0.045
ΔBrier	0.00164	[0.00043, 0.00294]	0.00251
FA raw slope	1.304	[-1.256, 3.884]	5.140
FA recalibrated slope	0.104	[-1.138, 1.477]	2.615
FA+GMM raw slope	0.372	[-2.181, 2.853]	5.034
FA+GMM recalibrated slope	0.093	[-1.238, 1.581]	2.819

Note. Differences are FA+GMM minus FA over the 24 common repeats. Calibration coefficients use 25 FA and 24 FA+GMM repeats and reproduce Table 4. Intervals are percentile intervals from 2,000 ordinary participant bootstrap samples with fixed predictions. Widths in this table are the differences between percentile limits; sample-size targets in Table S27 use normal-theory widths estimated from the simulated sampling standard deviations. AP, average precision; FA, factor analysis; GMM, Gaussian mixture model; ROC-AUC, area under the receiver operating characteristic curve.

Table S27. Evaluation Sample Sizes for Alternative Precision Targets

Estimand	Target full width	$\pi = 0.15$	$\pi = 24/103$	$\pi = 0.30$
ΔAP	0.10	103	103	103
	0.05	103[†]	103	103
	0.02	400→800	400→800	400→800
FA raw slope	0.80	3,200→6,400	3,200→6,400[†]	1,600→3,200
	0.40	12,800→25,600	12,800→25,600[†]	6,400→12,800
	0.20	$> 25,600$	$> 25,600$	$> 25,600$
FA recalibrated slope	0.80	800→1,600	800→1,600	800→1,600
	0.40	3,200→6,400	3,200→6,400	3,200→6,400
	0.20	12,800→25,600	12,800→25,600	12,800→25,600
FA+GMM raw slope	0.80	3,200→6,400	3,200→6,400	1,600→3,200
	0.40	12,800→25,600	12,800→25,600	12,800→25,600[†]
	0.20	$> 25,600$	$> 25,600$	$> 25,600$
FA+GMM recalibrated slope	0.80	800→1,600[†]	800→1,600	800→1,600
	0.40	6,400→12,800[†]	3,200→6,400	3,200→6,400
	0.20	$> 25,600$[†]	12,800→25,600	12,800→25,600

Note. $a \to b$ identifies the preceding tested size (a) and the first tested size meeting the target (b); sizes between them were not tested. An entry of 103 means the smallest tested size already met the criterion. An entry greater than 25,600 means no tested size met it. These are total evaluation sample sizes; expected positive counts are $N\pi$. Each cell uses 2,000 simulated samples under the specified fixed empirical outcome/prediction-vector distribution. Targets are approximate 95% interval full widths and are sensitivity-analysis settings, not clinical thresholds. † At least one boundary of the reported grid transition was within two Monte Carlo standard errors of its target; its grid classification is sensitive to Monte Carlo error. The simulation does not include model development or uncertainty in the working population.

At the observed positive proportion, the AP-width target of 0.02 was first met at 800 participants: the simulated width was 0.0166 (Monte Carlo standard error, 0.00027). The corresponding width at 400 was above 0.02. For a slope-width target of 0.40, the first passing sizes were 25,600 for both raw models and 6,400 for both recalibrated models. For the latter, simulated widths were 0.310 and 0.334 for FA and FA+GMM, respectively, with Monte Carlo standard errors of 0.00485 and 0.00563. The finer target of 0.20 for either raw slope was not attained at any tested size.

The contrast between AP and calibration precision identifies the calibration slope as a particularly demanding evaluation target for these narrow score distributions. These projections assume that the score distributions remain unchanged; they do not imply that the same sample sizes would be needed after a different model-development procedure.

Interpretation

These calculations describe evaluation precision if the specified joint outcome/score distribution persists. They hold the learned representations, prediction models, tuning choices, recalibrators, and split assignments fixed. They do not propagate estimation error in the working population, dependence between participants induced by overlapping training sets, or changes in prediction quality as training sample size increases. The projected numbers are therefore scenario-specific evaluation sample sizes, not requirements for developing the complete FA–GMM pipeline or evidence that 103 participants were adequate. Repeated simulation increases numerical precision, not the amount of independent clinical information in the observed cohort.

S11. Pediatric Outcome-Threshold Sensitivity

Methods and estimation status

This post hoc analysis retained the adult PHQ-9 threshold of 10 and applied a threshold of 11 in the pediatric stratum. The cohort remained 103 participants (83 adults and 20 pediatric participants), with 22 screen-positive participants (15 adults and seven pediatric participants). The corresponding no-skill AP baseline was 22/103 = 0.214. The prespecified threshold-10 analysis was retained as the primary analysis.

We refitted six models: the design-only benchmark, FA and native-scale FA+GMM with three representations (openSMILE, HuBERT, and PyFeat), FA and native-scale FA+GMM with four representations (additionally including text), and the four-representation standardized-input FA+GMM model. All models retained the cohort indicator. The original 25 repeats, five outer folds, and four inner folds were reused without restratification under the new labels; all training and evaluation partitions contained both classes. Preprocessing, FA, GMM, and penalty selection were repeated within the training splits. The random-seed bank, 25-value penalty grid (10^{-4} to 10^{2}), and initial optimizer settings (maximum 10,000 iterations; tolerance 10^{-8}) were unchanged. Following Section 14.1 of the statistical analysis plan, a ridge convergence warning prompted a check for zero-variance or near-constant columns and one retry with a maximum of 20,000 iterations, retaining the same design matrix, outcomes, split, penalty, solver, and tolerance. GMM-input standardization followed Section S6b. The input feature vectors and participant split assignments were held fixed.

All 750 planned model–outer-fold units were attempted. One three-representation FA+GMM unit failed because the logistic regression did not converge at penalty $\lambda = 10$ in inner fold 3 of repeat 20, outer fold 3, including after the specified retry. All input values were finite, and no zero-variance or near-constant columns were detected. Both attempts terminated abnormally after 10 optimizer iterations, before reaching either iteration limit. Its 21 test-participant predictions were retained as `not_estimated`; no alternative fit was substituted. Accordingly, this model's summary and paired comparisons used 24 complete repeats. The other five models used all 25 repeats. Model-specific means used each model's complete repeats, whereas paired means used only repeats evaluable for both models. Post-processing recalibration followed the original scoring procedure.

Table S28. Model Performance under the Alternative Pediatric Threshold

Model	Repeats	Raw AP	Raw ROC-AUC	Raw Brier	Recalibrated Brier
Design-only	25	0.217	0.513	0.1695	0.1704
FA, three representations	25	0.255	0.527	0.1679	0.1730
FA+GMM, three representations	24	0.238	0.496	0.1685	0.1726
FA, four representations	25	0.234	0.495	0.1684	0.1716
Native-scale FA+GMM, four representations	25	0.220	0.473	0.1688	0.1705
Standardized-input FA+GMM, four representations	25	0.232	0.487	0.1687	0.1737

Note. Entries are means of the metric calculated separately within each complete repeat. All models used the same 103 participants and alternative outcome definition. The three-representation FA+GMM model excluded repeat 20; its FA comparator's model-specific mean retained all 25 repeats. The design-only model used the adult-versus-pediatric cohort indicator. Recalibrated Brier scores describe the auxiliary shared-outer-CV post-processing procedure, not a fully nested calibration evaluation. AP, average precision; FA, factor analysis; GMM, Gaussian mixture model; PHQ-9, Patient Health Questionnaire-9; ROC-AUC, area under the receiver operating characteristic curve.

Paired comparisons and conditional uncertainty

Table S29 distinguishes two estimands. Panel A averages paired metric differences calculated within common evaluable repeats. Panel B first averages each participant's raw probabilities across common evaluable repeats and then calculates the paired metric difference. For Panel B, 2,000 outcome-stratified participant bootstrap samples (seed 20260407) retained 22 screen-positive and 81 screen-negative participants, and the 2.5th and 97.5th percentiles gave conditional 95% intervals. The same sampled participants were used for both models in each contrast. These intervals condition on the saved predictions and fixed split bank; the prediction pipeline was not refitted within bootstrap samples. No conditional intervals were calculated for the repeat-mean differences in Panel A.

Table S29. Paired Model Differences under the Alternative Pediatric Threshold

Panel A. Mean differences across common evaluable repeats

Comparison	Repeats	ΔAP	ΔROC-AUC	ΔBrier
FA+GMM minus FA, three representations	24	-0.0182	-0.0322	0.00069
Native-scale FA+GMM minus FA, four representations	25	-0.0146	-0.0212	0.00036
Standardized-input FA+GMM minus FA, four representations	25	-0.0023	-0.0073	0.00025
Standardized-input minus native-scale FA+GMM	25	0.0123	0.0139	-0.00012

Panel B. Differences from participant-mean probabilities [conditional 95% interval]

Comparison	ΔAP	ΔROC-AUC	ΔBrier
Native-scale FA+GMM minus FA, four representations	-0.0335 [-0.0944, -0.0088]	-0.0713 [-0.1150, -0.0303]	0.000409 [-0.000002, 0.000884]
Standardized-input FA+GMM minus FA, four representations	0.0041 [-0.0276, 0.0428]	0.0095 [-0.0410, 0.0584]	0.000078 [-0.000303, 0.000488]
Standardized-input minus native-scale FA+GMM	0.0376 [0.0111, 0.1104]	0.0808 [0.0275, 0.1397]	-0.000331 [-0.000966, 0.000273]

Note. Each difference is the first named model minus the second. Positive AP or ROC-AUC differences and negative Brier differences favor the first model. All Panel B contrasts use 25 common repeats of the four-representation models. Panel B point estimates and intervals do not estimate the repeat-mean contrasts in Panel A. The native-scale FA+GMM-minus-FA Brier interval includes zero; six decimal places retain its small negative lower limit. Intervals do not include uncertainty from refitting or alternative split banks. AP, average precision; FA, factor analysis; GMM, Gaussian mixture model; ROC-AUC, area under the receiver operating characteristic curve.

Interpretation

Under the alternative pediatric threshold, the native-scale GMM extensions had lower mean raw AP than their FA comparators in both representation sets. Input standardization attenuated the four-representation deficit, but the standardized-input model did not demonstrate incremental value over FA. The direction of its AP contrast differed between the two aggregation methods: -0.0023 for the repeat-mean difference and +0.0041 for the difference based on participant-mean probabilities, with the latter interval spanning zero. These results preserve the incremental-value conclusion while showing sensitivity of absolute performance and benchmark ordering to the operational outcome definition. Changing the cutoff does not establish criterion validity for the administered questionnaire in the pediatric age range, and the conditional intervals do not establish equivalence between representations.

References

[1] Muramatsu, K.; Miyaoka, H.; Kamijima, K.; Muramatsu, Y.; Tanaka, Y.; Hosaka, M.; Miwa, Y.; Fuse, K.; Yoshimine, F.; Mashima, I.; et al. Performance of the Japanese version of the Patient Health Questionnaire-9 (J-PHQ-9) for depression in primary care. *Gen. Hosp. Psychiatry* **2018**, *52*, 64–69. https://doi.org/10.1016/j.genhosppsych.2018.03.007.

[2] Harman, H.H.; Jones, W.H. Factor analysis by minimizing residuals (minres). *Psychometrika* **1966**, *31*, 351–368. https://doi.org/10.1007/BF02289468.

[3] Kashihara, K.; Takanabe, T.; Kiuchi, K.; Umehara, H.; Irizawa, K.; Nakataki, M.; Numata, S.; Kang, X.; Yoshida, M.; Matsumoto, K. Constructing multimodal counseling dataset for depressive state and feature analysis. In *Proceedings of the 2024 8th International Conference on Natural Language Processing and Information Retrieval (NLPIR 2024)*; Association for Computing Machinery, 2024; pp. 388–394. https://doi.org/10.1145/3711542.3711602.

[4] Kiuchi, K.; Kashihara, K.; Takanabe, T.; Umehara, H.; Irizawa, K.; Nakataki, M.; Numata, S.; Kang, X.; Yoshida, M.; Matsumoto, K. Interpretable multimodal emotion recognition in counseling dialogues via factor analysis and Gaussian mixture modeling. OSF Preprints **2025**, preprint. https://doi.org/10.31219/osf.io/2udp8_v1.

[5] Matsumoto, K.; Kiuchi, K.; Umehara, H.; Nakataki, M.; Numata, S. Interaction-driven dynamic fusion for multimodal depression detection: A controlled analysis of gating and cross-attention under class imbalance. *Brain Sci.* **2026**, *16*, 366. https://doi.org/10.3390/brainsci16040366.

[6] Snell, K.I.E.; Archer, L.; Ensor, J.; Bonnett, L.J.; Debray, T.P.A.; Phillips, B.; Collins, G.S.; Riley, R.D. External validation of clinical prediction models: Simulation-based sample size calculations were more reliable than rules-of-thumb. *J. Clin. Epidemiol.* **2021**, *135*, 79–89. https://doi.org/10.1016/j.jclinepi.2021.02.011.
